# Reversion of aortic valve cells calcification by activation of Notch signalling *via* histone acetylation induction

**DOI:** 10.1038/s41392-025-02411-8

**Published:** 2025-09-18

**Authors:** Gloria Garoffolo, Silvia Ferrari, Sara De Martino, Emanuele Pizzo, Veronica Candino, Lavinia Curini, Federica Macrì, Boudewijn P. T. Kruithof, Alessia Mongelli, Magda Grillo, Nadia Fanotti, Pamela Fejzaj, Manuel Casaburo, Azizah Alanazi, Nina Ajmone Marsan, Feras Khaliel, Ahmed Alsulbud, Marco Agrifoglio, Gualtiero I. Colombo, Mattia Chiesa, Antonella Farsetti, Carlo Gaetano, Angela Raucci, Maurizio Pesce

**Affiliations:** 1 Centro Cardiologico Monzino, IRCCS, Milan, Italy; 2 Consiglio Nazionale delle Ricerche, (CNR)-IASI “A. Ruberti”, Rome, Italy; 3 Leiden University Medical Center, Leiden, the Netherlands; 4 Netherlands Heart Institute, Utrecht, The Netherlands; 5 Istituti Clinici Scientifici Maugeri, IRCCS, Pavia, Italy; 6 Center for Translational and Experimental Cardiology (CTEC), University Hospital Zurich, University of Zurich, Zurich, CH Switzerland; 7 Department of Cell Biology, King Faisal Specialist Hospital & Research Center, Riyadh, Saudi Arabia; 8 Università degli Studi di Milano, Milan, Italy; 9 Turin School of Engineering, Turin, Italy; 10 College of Medicine, Alfaisal University, Riyadh, Saudi Arabia

**Keywords:** Cardiology, Senescence

## Abstract

Calcification of the aortic valve is a prevalent cardiovascular pathology in the aging population. Traditionally linked to inflammation, lipid accumulation, and risk conditions, this disease remains poorly understood, and effective treatments to halt its progression are not yet available. We hypothesized that calcification of the human valve interstitial cells (VICs) is associated with cellular senescence and alterations in the epigenetic setup, like in arteries. To verify this hypothesis, we examined the epigenetic marks (DNA methylation; Histones H3/H4 acetylation/methylation), the senescence and the calcification process in human VICs obtained from two distinct pathologic settings of the aortic valve (valve insufficiency and valve stenosis), and employed a mouse model of vascular/valve calcification, based on the administration of Vitamin D. Our findings revealed a link between the senescent phenotype of human VICs and calcification, characterized by increased DNA methylation and changes in histone epigenetic marks. To reverse the senescent/calcific VICs phenotype, we used Pentadecylidenemalonate-1b (SPV106), which activates KAT2B/pCAF histone acetyltransferase. In human VICs, SPV106 restored Histone acetylation marks, modified general chromatin accessibility and upregulated expression of Notch1, a potent inhibitor of valve calcification. The treatment also prevented the accumulation of calcific lesions in an ex vivo model of aortic valve calcification. In vivo treatment with SPV106 reduced calcification of the valve induced by administering Vitamin-D and positively preserved the valve motion compromised by calcification and the overall cardiac function. Based on these results, we propose the treatment with activators of histone acetylates as a viable option to prevent senescence/calcification of aortic VICs *via* restoration of correct chromatin acetylation, with concrete hopes to retard the progression of valve stenosis, a still largely unmet therapeutic need.

## Introduction

The progression of calcific aortic valve disease (CAVD) is associated with pathology markers and risk conditions in common with other cardiovascular disorders like atherosclerosis and coronary artery disease.^1^ Calcification of the aortic valve (AoV) depends primarily on the conversion of the cells enclosed into the valve leaflets matrix, the valve interstitial cells (VICs),^2^ into calcium depositing cells, causing the cusps to lose their mechanical integrity and elasticity. Factors such as mechanical stress,^3–7^ lipid accumulation^8^ and paracrine/inflammatory signalling,^9,10^ concur with the pathologic transformation of aortic VICs phenotype. Despite intense research to clarifying the sequence of cellular and molecular events determining pathological transformation of the aortic VICs, there are not yet effective solutions to block or retard the progression of the pathology.^1,11^ This situation leaves no other options than to replace the valve with prostheses.

Although VICs calcific differentiation involves several molecular effectors (e.g. transcription factors) in common with the calcific cells in bones (the osteoblasts), the formation of calcium nodules in the valve tissue has exclusive features resembling the vascular calcification process,^12^ characterized by secretion of mineralized micro-particles progressively altering the mechanics and the composition of the surrounding matrix.^5^ Given that arterial calcification involves the accumulation of senescent cells and concomitant modification of the epigenetic machinery,^13–15^ we were interested in extending this concept to calcification of the aortic valve. To this aim, we investigated the calcification of the valve in mice treated with high levels of vitamin D - a model of senescence-dependent aortic calcification^16^ - and compared the senescence/calcification potential of human VICs from valves with CAVD (sVICs) with that of VICs obtained from patients with insufficient valves (iVICs), which in general exhibit lower calcification levels.^7^ We also found that senescence/calcification of human sVICs correlated with higher DNA methylation and lower levels of histone Lysine tails acetylation. An estimation of the epigenetic age of sVICs and iVICs, as assessed by analyzing the methylation of *ELOVL2* gene promoter CpG islands^17^ indicated, finally, a trend of sVICs to be more consistently senescent compared to iVICs.

To reduce the epigenetic-driven senescence/calcification in human VICs, we treated sVICs with Pentadecylidenemalonate-1b (SPV106), a drug known to inhibit KAT3B/p300 and to activate KAT2B/pCAF histone acetylases^18^ already validated for reversion of hyperglycaemia-induced senescence in cardiac stromal cells.^19^ SPV106 reduced the sVICs calcification level, inhibited senescence markers, restored (at least in part) cell growth, and reverted the histone acetylation setup similar to that observed in iVICs. In line with these observations, SPV106 treatment restored expression of *Notch1* (expressed at lower levels in sVICs *vs*. iVICs), promoted nuclear translocation of NICD, and reversed expression of *Runx2* and *Sox9*, two crucial Notch1 targets involved in the control of valve pathologic process.^20^ This effect was mediated by a change in the general chromatin accessibility as revealed by the assay for transposase-accessible chromatin using sequencing (ATAC-seq)^21^ and an increase in chromatin acetylation of the Notch1 and Sox9 proximal promoter regions by chromatin immunoprecipitation (ChIP)^22^ assay. The SPV106 effects were finally confirmed in an ex vivo model of mouse AoV calcification,^23^ where addition of the drug inhibited formation of calcific nodules and expression of calcification markers,^16^ and the in vivo model of vitamin D-mediated AoV calcification, in which SPV106 prevented the decline in valve and cardiac function.

With this report, we propose a new mechanism for aortic valve calcification involving increased senescent phenotype of valve interstitial cells connected to reduction of Notch1 signalling. We also establish a relationship between senescent VICs phenotype and variations in epigenetic markers such as DNA methylation at age-sensitive hotspots and histone post transcriptional modifications, resulting into different global chromatin accessibility. We finally propose increasing of Histones acetylation by pCAF histone acetyl-transferases as a strategy to reduce senescence and calcification of human VICs and as an in vivo treatment to reduce the functional impact of calcification on valve motion and functionality.

## Results

### High vitamin D doses are related to senescence and calcification of the aortic valve

**Fig. 1:**
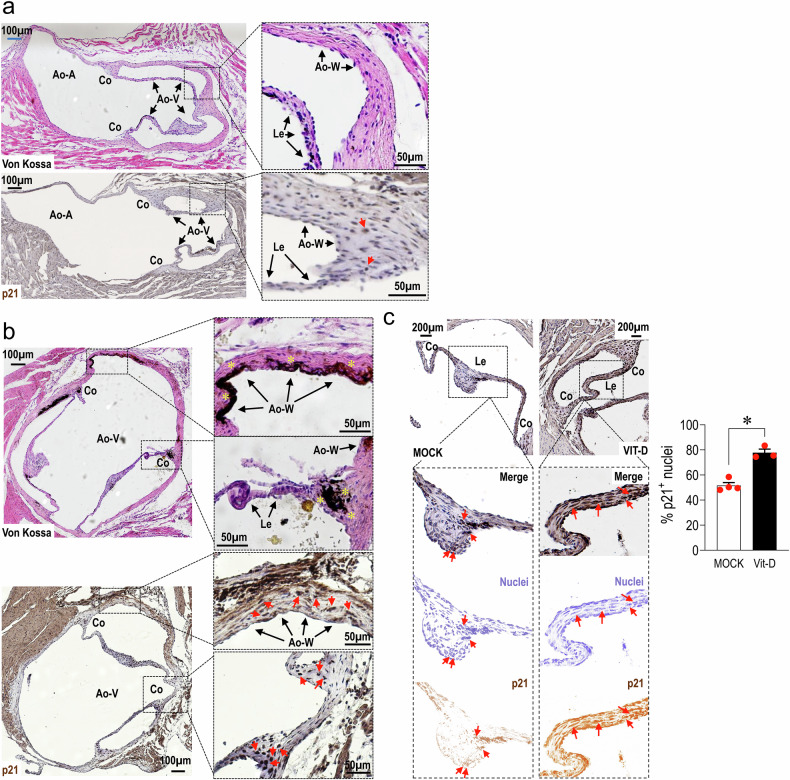
Assessment of aortic valve senescence and calcification in the region of the aortic valve (Ao-V) in control (**a**) and vitamin D-treated mice (**b**) by von Kossa staining and p21 immunohistochemistry. It is evident in panel **b** the presence of large calcifications in the aortic wall (Ao-W) and on the leaflets (Le), especially at the level of the commissures (Co). Note in panel (**b**) the presence of p21^+^ cells in the areas containing calcifications. **c** Quantification of nuclear-localized p21in the leaflets of vehicle and Vitamin D injected mice, as performed by image J. The nuclear staining of p21 was separated from the nuclear staining of Haematoxylin and quantified as number of p21^+^ nuclei in the leaflets (Le). Data are graphed as mean ± SE (individual data are represented by red circles), and statistically compared by unpaired t-test

Recent clinical evidences showed that patients treated with vitamin D and calcium supplementation is associated with a higher propensity of the aortic valve to calcify.^24^ Furthermore, previous work in our laboratories showed that treating mice with a high dose of vitamin D determines a rapid acceleration of an inflammation-independent calcification in the aorta through a senescence-dependent mechanism.^16^ We therefore analysed the AoV of mice receiving high vitamin D dose. Compared to mice treated with the mock solution (Fig. 1a), the Vitamin-D treated animals exhibited evident signs of calcification at the leaflet commissures, and in the wall of the valve annulus, corresponding to the Valsalva sinuses (Fig. 1b). The area of calcification exhibited an elevated number of interstitial cells with nuclear-localized p21^WAF1/CIP1^, one of the Cyclin-CDK inhibitors mediating cell cycle arrest and cellular senescence^25^ (Fig. 1b). The expression of p21 was also quantified in the leaflets by computer-assisted image analysis, revealing a generalized increase in the senescence of cells in the leaflets (Fig. 1c). These findings establish a direct correlation between calcification of the valve and cellular senescence, in analogy to what reported for arteries^16^.

### Increased senescence and calcification in stenotic vs. insufficient VICs

**Fig. 2:**
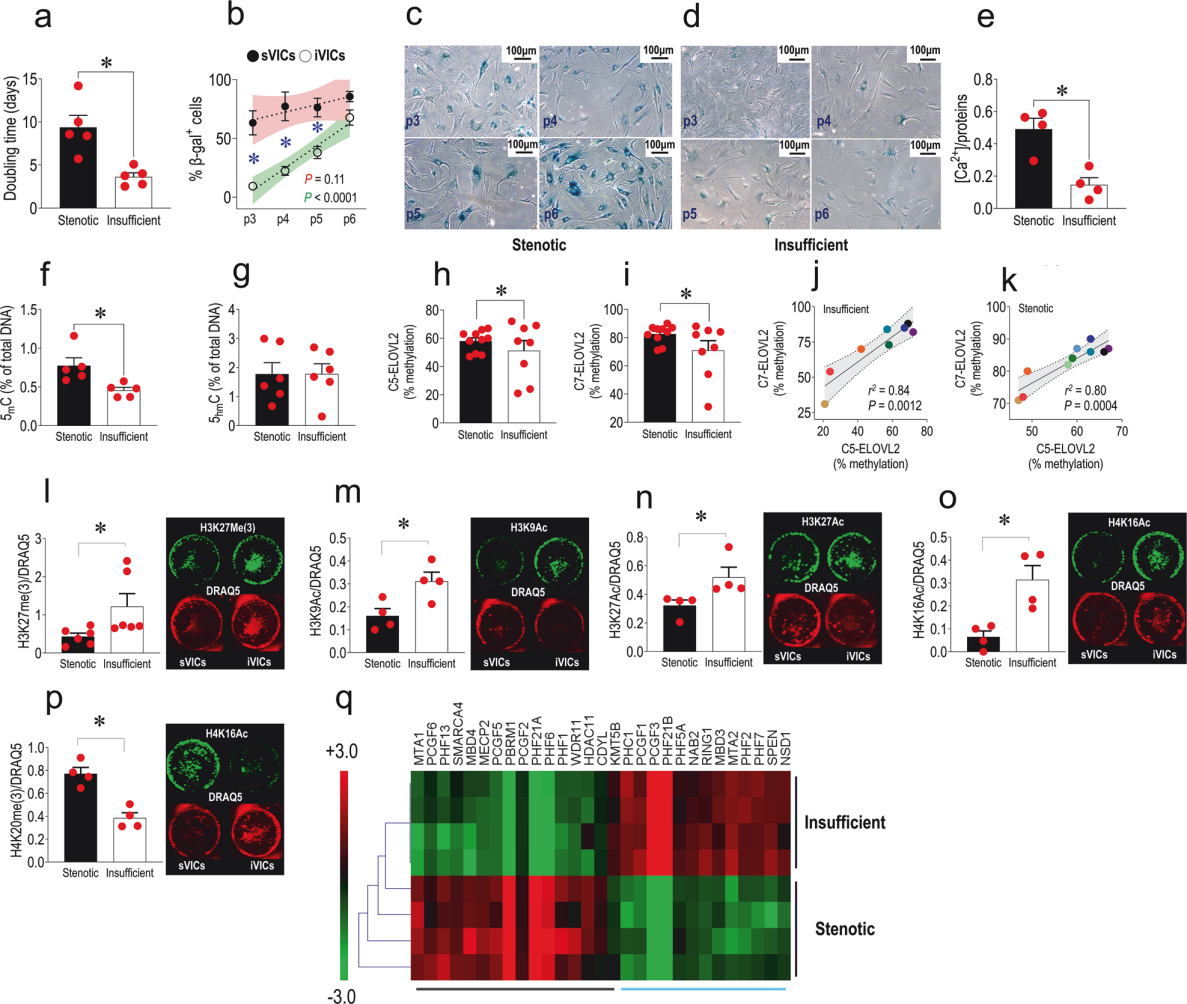
Comparison of replicative senescence in iVICs *vs*. sVICs as demonstrated by the increase in the doubling time between p1 and p2 (**a**) and β-Gal staining cells at passage numbers (p) between 3 and 6 (**b**–**d**). **e** sVICs also had an increased calcification potential compared to iVICs as determined by the assessment of intracellular calcium (normalized to cellular protein content), when cultured in a medium containing high calcium levels (cells all at p4). **f**, **g** Analysis of global DNA methylation/hydroxy-methylation in the two cell types showed an increased amount of methylated DNA (**f**) but not of 5’-hydroxymethylcytosine (**g**) (both expressed as percentage of total DNA) in sVICs compared to iVICs. **h**, **i** The methylation level of the C5 and C7 in *ELOVL2* promoter CpG islands was assessed by pyrosequencing after bisulfite conversion indicating a higher heterogeneity in iVICs *vs*. sVICs. **j**, **k** Linear regression analysis of the C5/C7 methylation ELOVL2 promoter sequences in VICs from insufficient compared to stenotic valves (r^2^ and *P* values of the two regression analyses are indicated in each plot). **l**–**p** Differences in the specific levels of selected Histones H3 and H4 methylation/acetylation marks in iVICS and sVICs, as detected by in-cell western analysis (representative images in insets). In each panel it is represented the quantification of each Histone modification normalized to the nuclear fluorescence intensity detected by DRAQ5 staining and representative images of the cells stained with each of the antibodies and DRAQ5 nuclear stain in the analysed culture wells. **q** Pathway-specific transcriptome arrays were used to assess the differential expression of transcripts involved in senescence, chromatin-remodelling and epigenetic regulation in iVIC and sVICs. The heatmap represents the results of an unsupervised clustering analysis of the differentially expressed mRNAs (genes listed in Supplementary Table 2). In all graphs, data are graphed as mean and SE (individual data are represented by red circles). Data were statistically compared by unpaired t-tests in plots (**a**, **e**, **f**, **g**, **l**–**p**), and * indicate *P* < 0.05. Data in (**b**) were compared by 2-way ANOVA, using a Bonferroni post-*hoc* analysis of the comparison between sVICs and iVICs senescence at the indicated passage numbers (* indicate *P* < 0.05 in the post-*hoc*). Data in (**h**) and (**i**) were analysed with F-tests to assess the variance of the biological age in sVICs and iVICs (* indicate *P* < 0.05). Data in panels (**j**) and (**k**) are individually represented as pairs of % methylation for C5 and C7 hotspots in insufficient and stenotic VICs. In each plot, the areas in colour indicate the 95% confidence intervals of the best-fit data interpolation (dotted straight line). The *n* of biological replicates is represented by the number of dots overlapped to each of the histogram plots, each indicating an individual cell donor

In order to find biological differences between human valve cells with different calcification potential, we employed cells from age-matched patients admitted to our hospital for substitution of aortic valves affected by terminal calcific stenosis or insufficiency, a valve pathology involving fibrosis and a low level of calcification^7^ (see Supplementary Table 1 for the main characteristic of these subjects). Cells were initially compared for their doubling time from passage 1 (p1) to passage 2 (p2). As shown in Fig. 2a, VICs from stenotic valves (sVICs) had a more extended doubling period compared to cells from insufficient valves (iVICs), and this was associated with a higher level of cellular senescence, as assessed by β-Galactosidase staining^26,27^ at each passage between p3 and p5 (Fig. 2b–d). The higher propensity of sVICs to calcify at these passages was confirmed by in vitro calcification assays (Fig. 2e, Supplementary Fig 1).

### sVICs senescence and calcification are epigenetically controlled

As shown in Supplementary Fig. 2, the higher senescence level and the increased propensity of sVIC to calcify did not depend on differences in the age of the donor individuals, the so called ‘chronological age’. Hence, we explored the possibility that the different senescence levels of the cells in the two pathologic settings are epigenetically programmed,^28^ such as in vascular stiffening and calcification,^29^ and reflect the ‘biological aging’ acceleration.^30,31^ To this aim, we assessed the level of global DNA methylation and we measured the methylation level of the C5 and C7 CpG islands within the promoter sequence of *ELOVL2* - a gene associated with biological aging.^17^ While the analysis of the global DNA methylation revealed an increase in the 5’-Methylcitosine content in sVICs compared to iVICs (5mC; Fig. 2f), not compensated by variations in the level of the product of the 5mC oxidation, the 5’-Hydroxymethylcitosine (5hmC; Fig. 2g),^32^ the analysis of the hotspots in the *ELOVL2* gene promoter revealed a higher variability of methylation in iVICs *vs*. sVICs (Fig. 2h, i). To exclude that the differences in the methylation level in the cells are due to stochastic variations, we performed linear regression analyses by plotting the data of C7/C5 methylation in each individual cell line from the two pathologic setting. As shown in Fig. 2j, k, the methylation of the two hotspots was consistently correlated, even if it was not correlated to the age of the donor individuals (Supplementary Fig. 3), again indicating a patient-specific programming of the cellular biological aging unrelated to chronological age.

Our attention was then drawn to histone modifications potentially characterizing the differences in senescence observed in sVICs *vs*. iVICs. In particular, we mapped modifications that are involved in a generalized increase in transcriptional activation (Acetylation of lysine-9 on histone H3 and lysine-16 on histone H4), repression (Methylation of lysine-20 on histone H3), and the so-called ‘poisoning’ for transcription (tri-methylation or acetylation of lysine-27 on histone H3).^33^ Panels l–p in Fig. 2 show the result of this analysis, clearly indicating an excess of activating modifications in iVICs (e.g., H3K9Ac, H4K16Ac) and the presence of an elevated level of specific repressor marks such as H4K20me(3), possibly indicating an enhanced amount of inactive, but with potential to be activated, chromatin. All these effects, again, were independent of the donors’ age (Supplementary Fig 4). To verify whether sVICs differ from iVICs for expression of epigenetic-active enzymes and chromatin remodelling factors, we analysed the expression of genes encoding for epigenetic writers/readers involved in chromatin structure/activity using pathway-specific RT-qPCR arrays and RNAs extracted from iVICs and sVICs, both at p3 and age-matched (Supplementary Fig 5). The results of unsupervised hierarchical clustering evidenced a differential expression of 29 genes (Supplementary Table 2), encoding for factors involved in chromatin architecture with specific functions in gene repression (e.g., *RING1, PHC1*, and *PCGF*- members of the PRC1 Polycomb repressor complexes^34^), histone H3 tails mark reading (e.g., *PHF1, -2, -6, -7, -21A/B*^35^), and SWI/SNF complex function (e.g., *SMARCA4/BRG1, PBRM1*^36^) (Fig. 2q). It was important to note that genes such as *MECP2*, involved in silencing chromatin containing methylated CpG islands, or *HDAC11* encoding for a protein with histone and non-histone deacetylase activity^37^ were expressed at lower levels in iVICs compared to sVIC. Conversely, iVICs expressed higher NuRD components *MBD3* and *MTA2* (balanced by a higher expression of *MTA1* in sVICs),^38^ and *NSD1* a histone methyltransferase with specificity for H3K36Met(2) involved in the maintenance of active chromatin.^39^ Altogether, these results suggest a higher degree of active chromatin or chromatin poised for transcription in iVICs.

### SPV106 reduces sVICs senescence and calcification by remodelling chromatin accessibility and modulating Notch expression and function

**Fig. 3:**
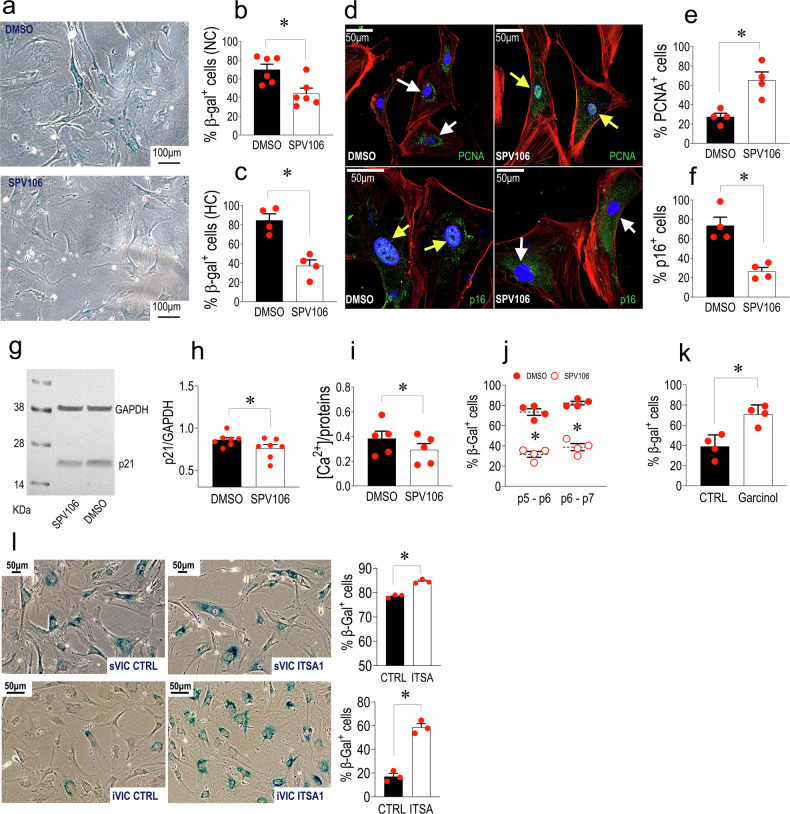
Treatment with SPV106 reduces senescence and calcification of sVICs mediated by Histone acetylation. **a** Reduction of β-Gal in cells treated for 7days with SPV106. Control treatment was performed using the same dilution of DMSO (the diluent of SPV106). **b**, **c** Quantification of the β-Gal^+^ cells treated and untreated with SPV106 under normal (NC) or high calcium (HC) concentrations: in both cases the epigenetic drug reduced the level of cellular senescence. **d** PCNA (top) and p16 (bottom) immunofluorescence in DMSO and SPV106-treated sVICs. White and yellow arrows indicate, respectively, cells with cytoplasmic or nuclear localization of the two markers. Note the variation in the localization of the two antigens in control *vs*. SPV106-treated cells. Red fluorescence in all panel represents stress fibres as detected by phalloidin-TRITC staining. **e**, **f** Quantification of results indicated the effect of SPV106 in increasing the percentage of cells in active phases of the cell cycle (PCNA^+^) and a reduction of the nuclear expression of the senescence marker (p16^+^). **g**, **h** Western analysis and relative quantification of p21 senescence markers expression in DMSO and SPV106-treated cells. **i** Assessment of the sVICs normalized calcium concentration when cultured in the presence of calcification medium, showed a significant reduction in the presence of SPV106. **j** The effects of SPV106 on reduction of sVICs senescence was maintained for two passages (p5 → p6 and p6 → p7) after withdrawal of the drug. **k** Treatment of iVICs with Garcinol, an inhibitor of HAT, determined a significant elevation of the β-Gal^+^ cells in the culture, confirming the importance of histone acetylation activity for senescence process in human VICs. **l** Similar effects were obtained using ITSA, an HDAC activator. Interestingly, the effect of the drug was more potent on iVICs (graph on the bottom) than in sVICs (graph on the top). In all graphs, data are represented as mean ± SE. * indicate *P* < 0.05 by paired t-test. The *n* of biological replicates is represented by the number of dots overlapped to each of the histogram plots, each indicating an individual cell donor

Previous work showed that cells induced to become senescent by metabolic stress could be reverted to a pre-senescent status with recovered growth by employing a drug (Pentadecylidenemalonate-1b; SPV106), inducing genome-wide histone acetylation by inactivation of p300/CBP and activation of GCN5/pCAF histone acetylases.^18,19^ We thus set *an* in vitro treatment protocol with this drug to reduce senescence in sVICs. After setting 15 µM as the most effective SPV106 concentration in reducing the level of β-Gal^+^ cells at p5 (Supplementary Figs. 6, 7; Fig. 3a–c), the drug was used to assess expression of PCNA and p16 (Fig. 3d–f), the expression of p21 (Fig. 3g, h), and the intracellular calcium accumulation (Fig. 3i; Supplementary Fig 8). As shown in the figures, the treatment significantly reduced the senescence markers and the calcification in sVICs. A pulse-chase experiment performed by treating sVICs with SPV106 at p5 followed by drug washout until p7 clearly showed that the senescence-inhibiting function of the drug lasted for at least two passages (Fig. 3j). Finally, confirming the relevance of histone acetylation for the control of VICs senescence process, treating cells with 20 µM Garcinol, a broad HAT inhibitor^40^ and ITSA-1 an HDAC activator^41^ increased senescence level in iVICs and, at a lower level, in sVICs (Fig. 3k, l).

**Fig. 4:**
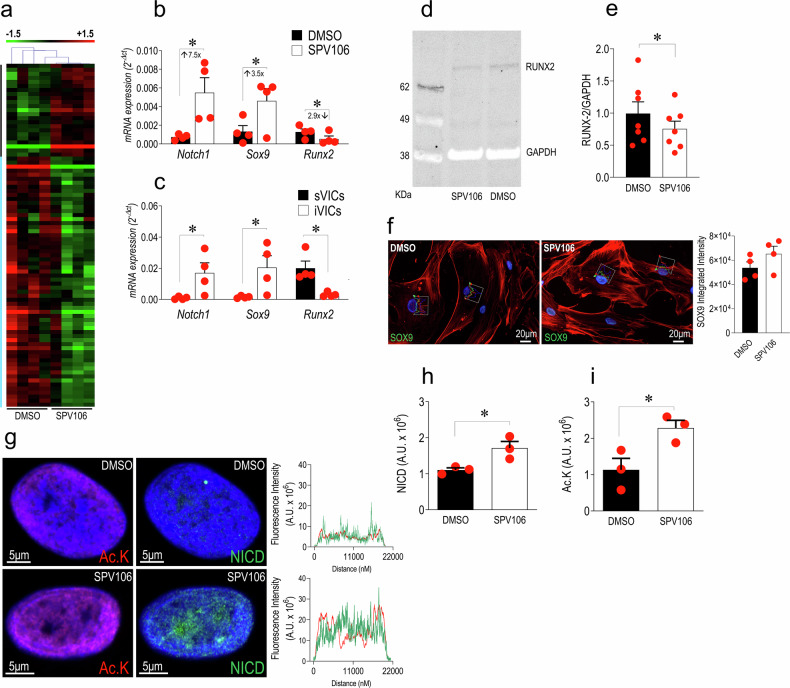
Variation in the expression of epigenetic regulators in response to treatment of sVICs with SPV106. **a** Heatmap from an unsupervised clustering analysis of the genes significantly modulated by SPV106 in sVICs (gene listed in Supplementary Table 3) showing extensive expression changing of transcripts encoding for epigenetic modulatory and chromatin-associated factors caused by the drug. **b**, **c** Expression of *Notch1* and the key genes (*Runx2/Sox9*) controlled by Notch pathway in sVICs (±SPV106 treatment, (**b**) and in sVICs *vs*. iVICs (**c**). In stenotic VICs, (black bars), high levels of *Runx2* pro-osteogenic transcription factor correlated with low levels of *Notch1* and *Sox9*, while in iVICs (white bars) the situation was opposite, with elevated expression levels of *Notch1*/*Sox9* and low levels of *Runx2* transcripts. Treatment of sVICs with SPV106 reverted the expression of these genes at a level similar to that observed in iVICs, consistent with a lower propensity to calcify. **d**, **e** The decrease of Runx2 by SPV106 was in effect also at a protein level as shown by Western analysis and the relative quantification. **f** Sox9 upregulation was also observed at protein level with an increase in nuclear localization, as quantified by integration of nuclear fluorescence (see graphs overlapped to individual nuclei). **g** SPV106 increases the overall level of lysine acetylation in chromatin and of NICD in the nuclei of the treated sVICs. The images on the top of the panel show the high resolution confocal microscopy images of the cells stained with anti-*pan*-acetyl-Lysine (red fluorescence) or anti-NICD (green fluorescence) antibodies (plus DAPI – blue fluorescence – as a staining of the nuclei), and the fluorescence profile of a typical control (top right) or SPV106-treated cell (bottom right; note the difference in the red and green fluorescence intensity in the CTRL vs. SPV106-treated cells). **h**, **i** Quantification of the fluorescence data, from which it emerges a positive effect of SPV106 on increase in acetylation of nuclear proteins and NICD nuclear accumulation. In all graphs data are represented as mean ± SE. * indicate *P* < 0.05 by paired t-test. The *n* of biological replicates is represented by the number of dots overlapped to each of the histogram plots, each indicating an individual cell donor

Pathway-specific transcriptomics was performed on RNAs extracted from sVICs treated or not with SPV106, using low-density RT-qPCR arrays. The treatment with SPV106 determined a significant change in the expression of 85 of the 243 profiled genes (~35%; Supplementary Table 3), with a total of 23 genes upregulated and 62 downregulated, and a clear separation between genes up/down-modulated by unsupervised clustering (Fig. 4a). Supplementary Table 4 lists the most significantly enriched pathways indicating up- and downregulated genes. Pathways with a functional annotation on chromatin modification, remodelling, and organization exhibited upregulated genes in common such as *EED*, *KAT8*, and *KMT2E*, encoding, respectively, for a component of the PRC2 complex engaged in H3K27 mono-di-tri-methylation,^42^ for a Lysine-acetylase important for H4K16 acetylation,^43^ and H3K4 methylation reader important in cell cycle progression and chromatin stability.^44^ They included series of downregulated genes involved in DNA methylation (*DNMT1, DNMT3A*),^45^ histone deacetylation (*HDAC1, HDAC10*),^46^ and other classes of chromatin remodelling factors, mostly associated with chromatin reading, accessibility, and remodelling factors (i.e., *BR, PF1*, *BRPF3*, *CHD4, DOT1L, ING5, SUV39H1*).^47–51^ Another set of enriched pathways suggested implication of SUMOylation in chromatin remodelling and cellular senescence. These pathways were characterized by downregulation of *CBX4*, encoding for SUMO E3 ligase, and various Polycomb PRC1 repressor complex (*CBX6, CBX8, PCGF2, PHC2, RING1)*.^52^ In these pathways, it was interesting to note the upregulation of *BMI1*, establishing a possible link between a possible reversion of chromatin silencing by SPV106 and reduction of cellular senescence due to DNA damage response characterized by CBX4-mediated *BMI1* SUMOylation.^53^ The functional genomics analysis also clearly indicated the involvement of pro-survival pathways such as those relative to *PI3K/AKT/PTEN* and *P53* expression, with concomitant upregulation of *PIK3CA* (the gene encoding for the 110 kDa catalytic subunit of PI3K) and the PIP3 signalling antagonist *PTEN*.^54,55^ They were also characterized by regulation of *CARM1, GADD45A, E2F1* and *ING2/5*, all involved in modulating the TP53-dependent senescence pathway.^56,57^ It was also interesting to observe the enrichment of two senescence-related pathways characterized by concomitant upregulation of *BMI1* and *CDKN2A* gene (encoding for p16^INK4/ARF^). Given the direct function of BMI-1 in repression of the p16 promoter,^58^ these results are consistent with an increase in cellular self-renewal in cells treated with SPV106.^59,60^ Finally, six pathways emerging from the informatics search indicated the implication of the Notch1 signalling in the possible regulation of sVICs phenotype after treatment with SPV106. These pathways were characterized by downregulation of histone deacetylase *HDAC1/7/10* genes and Lysine Acetyl-transferase 2A (*KAT2A*), suggesting a de-repression of Notch1 activity potentially inhibiting sVICs osteogenic programming^61,62^ (Supplementary Fig. 9). This hypothesis was verified by investigating the expression of *Notch1* and of *Runx2* and *SOX9*, two Notch1-dependent regulatory master genes directly involved in AoV calcification.^20^ Data validation by RT-qPCR, western analysis and immunofluorescence showed an evident upregulation of *Notch1* and *Sox9* mirrored by *Runx2* downregulation in the total RNA of sVICs treated with SPV106 (Fig. 4b). This expression pathway characterized by high levels of *Notch1* and *Sox9* and low levels of *Runx2* also emerged in the sVICs *vs*. iVICs comparison (Fig. 4c), suggesting, in line with data in the literature,^61,63^ that repression of senescent/calcific phenotype in human VICs by SPV106, is associated to elevated levels of *Notch1* and *Sox9* transcripts and inhibition of *Runx2*, a phenotype reminiscent of iVICs. Finally, treatment of sVICs with SPV106 induced a reduction in Runx2 protein expression (Fig. 4d, e), and an increase in nuclear localization of Sox9 (Fig. 4f), and of the transcriptionally active portion of Notch protein (the so-called Notch intracellular domain, NICD) whose transcriptional effects are enhanced by HAT-dependent acetylation^64^ (Fig. 4g–i). This evidence suggests a multilevel control of the valve calcification process, involving transcriptional upregulation of *Notch1* and transcriptional activation of NICD.

**Fig. 5:**
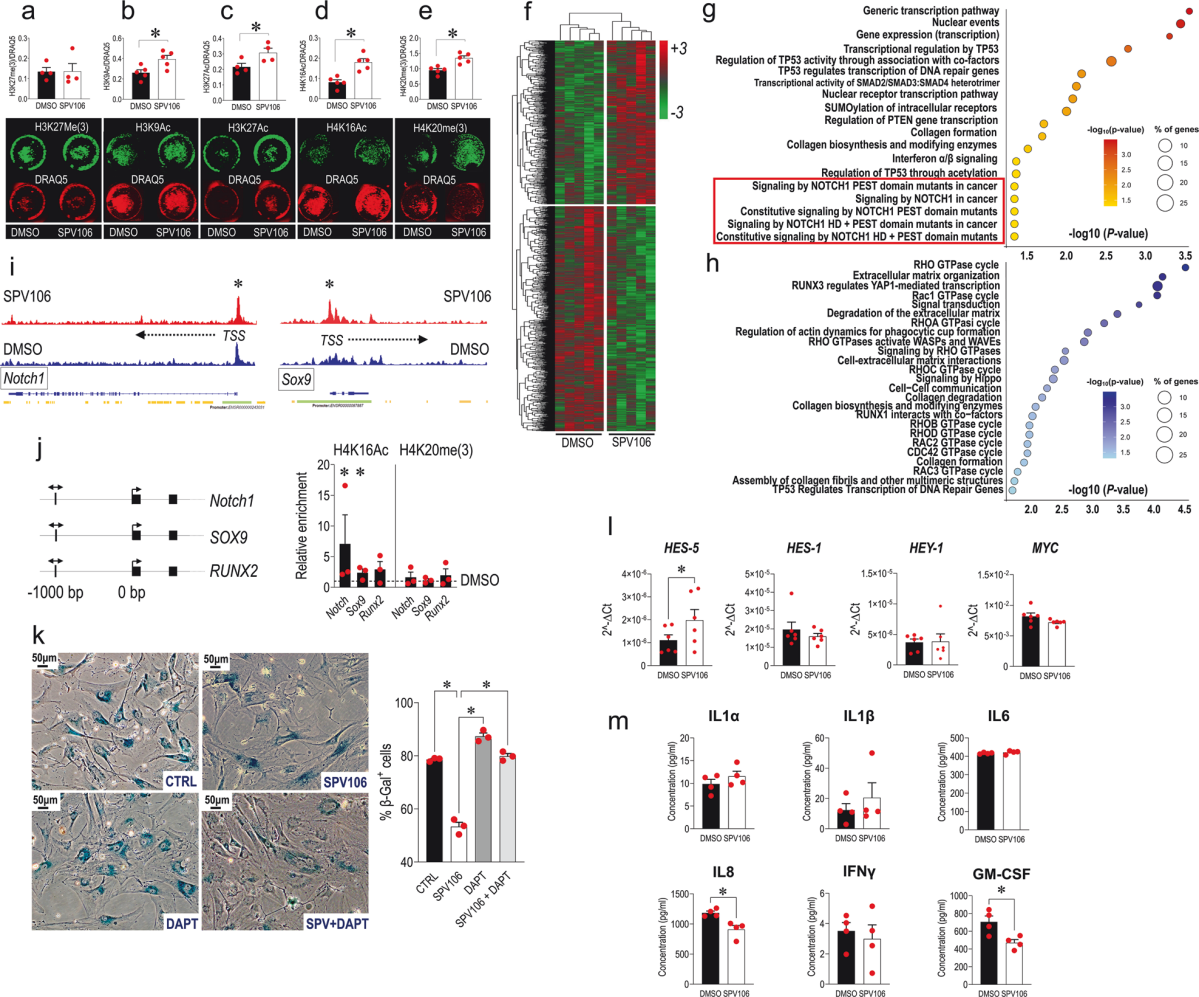
SPV106 restores at least in part the Histones H3/H4 epigenetic setup in sVICs. Panels **a**–**e** show the levels of each histones H3/H4 modifications (normalized to the total cell number by DRAQ5 nuclear staining) in control sVICs and sVICs treated with SPV106, as detected by in-cell Western analysis. With the exception of the tri-methylation on the Lysine 27 on histone H3, SPV106 increased the histones H3/H4 acetylation and the tri-methylation on Lysine 20 on histone H3. **f** Heatmap representing the unsupervised clusterization of the genes with more open (red) or more closed (green) chromatin emerging from ATACseq of DMSO or SPV106-treated sVICs (normalized data in Online XLS file c and category of genomic sequences described in Supplementary Fig 11). **g**, **h** Bubble plot representation of Reactome Pathways encompassing genes with more open or more closed chromatin configuration, and therefore potentially more transcriptionally active or repressed, respectively. Pathways are vertically represented in order of decreasing significance (-Log *P*-value indicated along the *x* axis) and bubbles dimension is proportional to the amount (%) of the genes represented in each pathway with the indicated functional annotation. Note in (**g**) (encircled) the presence of 5 pathways with more open chromatin configuration related to Notch signalling. **i** Graphic representation of the reads distribution in the region of the *Notch1* and *Sox9* promoters as detected by ATACseq. Note the presence of peaks with a higher number of reads in the region close to the transcription start site (TSS) in a representative distribution in chromatin from DMSO *vs*. SPV106-treated cells. **j** Chromatin immunoprecipitation was performed using antibodies specific for human H4K16Ac or H4K20(me)3 followed by qPCR. The representation on the left shows the positioning of the primers on the *Notch1*, *Sox9* and *Runx2* gene promoters at -1000bp upstream of the TSS. The graph on the right shows the quantification and the statistical analysis of the enrichment experiment by qPCR. From these results, it is possible to conclude that H4K16Ac (but not H4K20me(3)) modification determines a significant increase of the chromatin accessibility in the *Notch1* and *Sox9* promoter, justifying the increased expression observed in SPV106-treated cells. The dotted bar in the graph indicates the relative enrichment of the chromatin in DMSO-treated cells equalized to 1 for a comparison with the level observed in SPV106-treated cells, represented by the bars in the histogram plots. **k** The activity of the Notch pathway in rescuing the senescent phenotype of sVICs was validated using DAPT, a specific inhibitor. As shown, DAPT inhibited the effect of SPV106 when added to cells in combination. **l**, **m** The treatment with SPV106 was finally validated by monitoring the transcriptional effect on Notch-reported transcriptional targets *HES-5*, *HES-1*, *HEY-1* and *MYC*, as well as on secretion of SASP cytokines. In all graphs data are represented as mean ± SE. * indicate *P* < 0.05 by paired t-test. The *n* of biological replicates is represented by the number of dots overlapped to the histogram plots, each indicating an individual cell donor

The variation in the expression of chromatin remodelling factors observed in cells treated with the pCAF/KAT2B activator, suggested significant effects on chromatin accessibility and global genes expression. To substantiate this hypothesis mechanistically, we analysed the Histones epigenetic marks discriminating iVICS and sVICs (Fig. 2l–p) in sVICs treated with SPV106. The results of in-cell Western analyses (Fig. 5a–e) showed, except for H3K27me(3), an apparent increase in the amount of the Histones H3 and H4 epigenetic marks. Interestingly, the increase in Histone epigenetic marks was not paralleled by a reduction of the DNA methylation or the methylation of the *ELOVL2* hotspots (Supplementary Fig 10). This result shows that the senomorphic action^31^ of SPV106 in restoring sVICs growth and inhibiting sVICs calcification occurs through a net increase in the histone epigenetic marks, but it does not involve DNA de-methylation.

We next investigated whether the increased epigenetic marks in the chromatin of sVICs treated with SPV106 were associated with variations in the general chromatin accessibility in regions associated with pathways connected to valve disease. To this aim, we performed ATAC-seq^65^ on chromatin extracted from sVICs treated or not with SPV106. After performing alignment of the reads to the GRCh38 human genome by BWA-mem,^65^ genomic regions with high levels of transposition/tagging events (i.e., those with high chromatin accessibility) were determined using the MACS3 peak calling algorithm.^66^ Then, we analysed the loci with significantly higher or lower chromatin accessibility in a pairwise DMSO- *vs*. SPV106-treated cells comparison, performing a differential analysis with the DaMiRseq^67^ (peak filtering and normalization) and LIMMA^68^ (statistical analysis) R packages, in which a paired test has been implemented. Of the 128,862 loci identified by ATACseq (Supplementary Data 1) a), 32,439 passed stringent quality filtering, namely more than 20 reads in all samples (Supplementary Data 1) b) Of them, 771 bore higher accessible, and 1060 lower accessible chromatin (Supplementary Data 1, c). Unsupervised clustering of the data showed a clear separation between genes with higher or lower chromatin accessibility due to treatment (Fig. 5f), with a percentage of 58.7 gene promoter/enhancers, 7.5 insulators and 7.9 intergenic regions (Supplementary Fig 11) In order to derive insights on the transcriptional pathways potentially affected by the epigenetic treatment, we next analysed the dataset of the genes with significantly different chromatin accessibility with Reactome.^69^ By this, we were able to identify 60 pathways containing genes with significantly increased chromatin accessibility and 74 pathways encompassing genes with less accessible chromatin conformation, suggesting, respectively, activated or repressed conditions. These pathways, listed in Supplementary Tables 5 and 6 are graphically represented in Fig. 5g, h. As shown, they include Notch1- and Rho-related pathways among those with a potentially positive or negative activation, respectively.

The increase in the opening of the chromatin in the promoter region of *Notch1* and *Sox9*, but not of *Runx2*, was evident by the analysis of the reads distribution (Fig. 5i and data not shown), thus providing a rationale for the higher mRNA levels observed for the two genes in SPV106-treated cells. To confirm that treatment with the epigenetic drug determined structural modifications in the promoters of these genes, we performed Chromatin Immunoprecipitation assay (ChIP) in sVICs treated with DMSO or SPV106 using specific antibodies recognizing acetylated lysine-16 and three-methylated lysine-20 on histone H4 (two of the significant histone marks restored by the treatment, Fig. 5d, e), followed by PCR-based quantification of enriched promoter sequences of the three genes. To do this we chose primers encompassing the region 1000 base pairs (bp) upstream (−1000) of the described transcriptional start site (TSS), as indicated by the ATACseq (Fig. 5i). The results of ChIP-qPCR indicated a positive enrichment of the H4K16Ac (but not of the H4K20me(3)) onto *Notch1* and *Sox9* promoters at −1000bp (Fig. 5j). Given that the highest H4K16Ac chromatin enrichment occurred on *Notch1*, we also analysed a wider portion of the promoter using PCR primers pairs encompassing the −1500 to 0 bp (TSS) genomic region. As shown in Supplementary Fig 12, the −1000 region was the only one that resulted significantly enriched by ChIP with H4K16Ac-specific antibody, identifying this site as the most sensitive to the histone H4 hyperacetylation resulting from SPV106 treatment. To finally validate the involvement of Notch signalling in reducing the sVICs senescence mediated by SPV106, we used DAPT, a specific inhibitor of the Notch pathway,^70^ alone or in combination with the epigenetic drug. Results showed that DAPT reverted the positive effect of SPV106 on sVICs senescence, suggesting a direct involvement of Notch in suppressing the sVICs pathologic phenotype. Furthermore, treatment with SPV106 inhibited the transcription of at least one of the tested Notch direct transcriptional targets (*HES-5*)^71^ (Fig. 5k) and reduced the secretion of two of the tested SASP cytokines IL8 and GM-CSF (Fig. 5l, m). By contrast, the drug did not affect the expression of the genes encoding for fibrotic genes TGFβ1 and its receptor TGFβ-R1 (Supplementary Fig 13), or of genes relevant for oxidative stress *NQO1*, *TXNRD1* and *PRDX4*^72^ (Supplementary Fig 14), showing the specificity of the treatment with SPV106 to reduce sVICs pathologic phenotype by targeting senescence/calcification and, at least in part, SASP phenotype, but not progression toward fibrosis.

### SPV106 prevents the formation of calcium deposits in the aortic valve preserving cardiac function

**Fig. 6:**
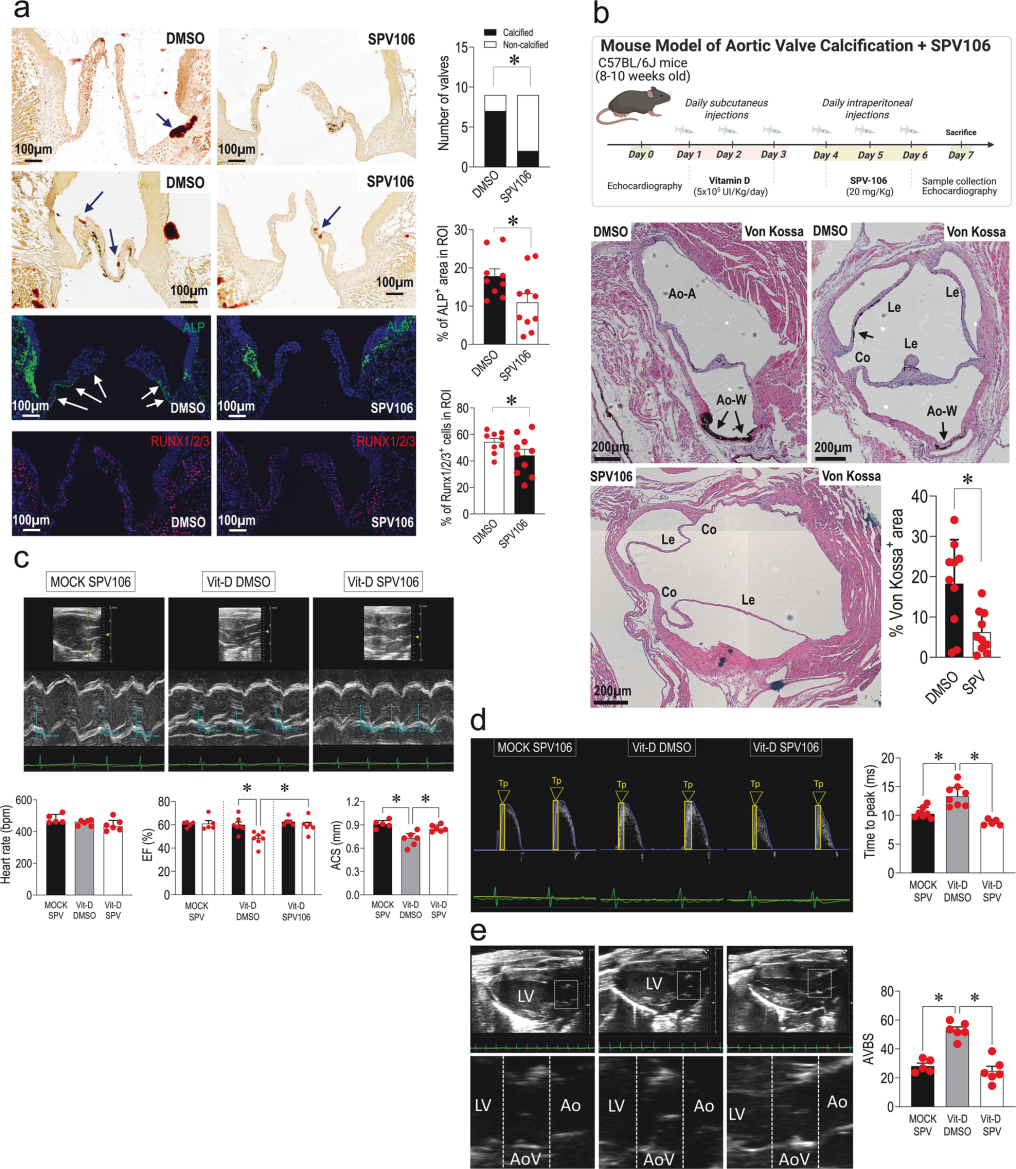
**a** Representative pictures of Alizarin Red, alkaline phosphatase (ALP) and Runx1/2/3 staining of mouse aortic valves cultured for one week in the MTCS under calcifying conditions in the presence of SPV106 or DMSO (control) showing the significant reduction in the number of calcified valves, the ALP-positive area and the percentage of RUNX1/2/3-expressing cells with SPV106 treatment. As shown in the bar graphs on the right, treatment with SPV106 reduced significantly the number of calcified valves and the percentage of cells expressing calcification markers compared to controls. Please refer to Supplementary Fig. 15 for a high magnification and an immunophenotype staining of cells of the valve region represented in the top left panel. **b** On the top of the panel the experimental scheme adopted to administer SPV106 to mice receiving high doses of Vitamin D is represented. After the initial administration of the Vit-D for three days, the mice were injected intraperitoneally with vehicle (DMSO) or SPV106 for three day before echo analyses and sacrifice (this image was realized with Biorender licensed to Centro Cardiologico Monzino, IRCCS). In the lower part of the panel are represented images of von Kossa staining of the aortic valves of the mice, in which it is evident the presence of large calcifications in the areas of the aortic wall (Ao-W) and the leaftets (Le), as indicated by arrows. SPV106 treated samples were free of large lesions, indicating the ability of the drug to reduce calcification. Quantification of the anti-calcific effect of SPV106 expressed as % of calcification areas in the control *vs*. treatment groups. **c** Echocardiogram sequences showing the opening/closing cycles of the heart in the Mock-treated, Vit-D/DMSO and Vit-D/SPV106 treated mice. As shown from the analysis of the aortic cusps separation (ACS) and the ejection fraction (EF), treatment with the drug reverted the detrimental effects of Vitamin D on valve motion and the overall function. Note in the histogram on the right that administration of the epigenetic drug prevented deterioration of the cardiac function as assessed by the pre- vs. the post-treatment indicated by the black and the open bars, respectively. **d** Analysis of time to peak (Tp), the time necessary to reach the max transvalvular flow at valve opening confirmed the restoration of valve motion by SPV106 compared to Vit-D/DMSO treatment. **e** Analysis of the echocardiographic aortic back scatter (AVBS) using a parasternal long-axis view. The valves are captured at diastole (valve closed). Note the difference between the echo sound in the MOCK and the Vit-D/SPV106-treated *vs*. the Vit-D/DMSO-treated animals. The AVBS quantification graph shows that treatment with SPV106 reduced valve calcification. * indicate *P* < 0.05 by Fisher exact test, unpaired/paired t-test or 1-way Anova with Bonferroni post-hoc. The *n* of biological replicates is represented by the number of dots overlapped to the histogram plots, each indicating an individual heart/animal

To validate the effects of SPV106 on valve calcification, we employed two complementary models. In an ex vivo model, we used a miniature tissue culture system (MTCS), designed to culture the aortic valve in its natural position in the heart, and expose it to pathologic hemodynamic conditions in the presence of inorganic phosphates, with DMSO or SPV106 used at the same concentration as in cell culture.^23,73–75^ In the second model, we employed the treatment protocol with Vitamin-D to assess the effects of SVP106 in vivo. To this aim, in line with our previous studies^76^ we used SPV106 at a 20 mg/kg concentration for three days after vitamin D injection (Fig. 6b). The results of the ex vivo model (Supplementary Fig. 15, Fig. 6a) showed a significant reduction of valves with detectable calcifications in the cusps and the aortic ring in the SPV treatment. In addition, quantifying immunofluorescence staining with antibodies specific for Alkaline Phosphatase (ALP) and RUNX1/2/3 showed a reduction of the osteogenic commitment in the valves treated with SPV106, even though reduction of calcification was not associated to variations in the level of p16^+^ and p21^+^ cells (Supplementary Fig. 16). An improvement of the aortic valve calcification was clearly observed in the in vivo model, in which the administration of the epigenetic drug reduced the presence of calcium deposits and of Osteopontin (OPN) a matricellular protein involved in bone deposition in the cusps and the aortic ring (Supplementary Fig. 17). By contrast, SPV106 did not affect the serum concentration of OPN, considered a clinical marker of aortic valve stenosis^77^ (data not shown), suggesting a tissue-specific and not a systemic effect.

Apart from the reduction of calcifications, SPV106 also had a beneficial effect on the overall cardiac function and valve motion, as detected by echocardiographic assessment. For example, the administration of Vitamin D caused a decrease in the ejection fraction, and this decline was prevented by administration of SPV106 (Fig. 6c). More directly on the valve function it was the effect of the drug on the cusp separation^78^ that was decreased by the Vit-D treatment (consistently with a more rigid structure) and preserved in mice receiving SPV106 (Fig. 6c), and the time-to-peak velocity^79^ that was delayed by the Vitamin-D and restored at the control level by the epigenetic drug (Fig. 6d). Interestingly, another parameter linked to aortic valve calcification, the so-called echocardiographic aortic valve back scatter (AVBS), directly referable to calcification,^80^ was also reduced by SPV106 treatment (Fig. 6e). Other clinically relevant valve functional parameters such as, e.g. the Vmax were not affected by the vitamin-D and the vitamin-D/SPV106 treatments, likely due to the early phases of valve calcification detectable by our protocol.

## Discussion

The search for novel treatments to retard or block the progression of CAVD is an intense research area addressing a critical and continuously growing clinical need. As outlined in various contributions focused on CAVD, until now the disease can be effectively treated only with valve replacement. At the same time, mechanistic studies have just begun to provide a picture of the integrated contribution of extrinsic (risk) conditions (i.e., altered metabolism, dyslipidaemia), intrinsic/extrinsic cellular components (valve endothelial/interstitial cells, inflammatory cells) and biomechanical factors (e.g., shear stress and stiffness sensing) involved in the onset and progression of the pathology.^11,81,82^ This uncertainty limits the availability of pharmacological treatments to retard or block this progression.^83^

### Stenotic and insufficient VICs exhibit crucial differences in the extent of epigenetically set senescence independently of donor chronological age

Mechanistically, valve interstitial cells calcification process has been for a long time assimilated to osteoblast-like differentiation, with the identification of common molecular pathways (e.g. the Runx2/BMP pathway).^84^ More recently, new results have highlighted the relevance of mechanical factors such as the cellular stress consequent to strain, and the response to pro-inflammatory signalling.^3,85,86^ In keeping with this evidence, nano-analytical methods have established peculiar differences between the mineralization in the bones *vs*. that of the valves, characterized by secretion of mineralized microparticles and similarities with the vascular calcification.^5,12^

Given the implication of senescence in vascular calcification, we hypothesized that VICs obtained from patients with different AoV pathologic settings (insufficiency and stenosis) may differ for the level of cellular senescence and epigenetic setups.^87–90^ Data presented in Figs. 1 and 2 support the hypothesis that sVICs calcification is associated with a reduced capacity of the cells to grow (the so called proliferative arrest^91^) related to a pre-established senescent status programmed before the beginning of the in vitro cell culture independently of the patient chronological age. This condition was absent in iVICs, which exhibited a consistently shorter doubling time and a reduced β-Gal expression. The higher expression of senescence markers in sVICs of subjects with comparable chronological age to that of iVICs donors, led us to test the possibility that differences in the epigenetic ‘clock’^92^ between the two populations may determine the variation in the calcification potential of the cells from the two pathologic settings. A first evidence supporting this conclusion emerged from the differences in the content of methylated DNA, showing around a double level of 5mC in sVICs *vs*. iVICs independently of donor ages (Fig. 2). Interestingly, the content of 5hmC, the first intermediate generated by TET enzymes during DNA demethylation process,^93^ was not different in sVICs *vs*. iVICs. This suggests that the increase in the 5mC in the two pathologic settings might derive from an imbalance in DNA demethylation due to differences in the availability of TCA cycle intermediates such as αKG/succinate, known to function as cofactors in TET-dependent DNA demethylation.^94^ A second indication that the epigenetic clock could be shifted in sVICs compared to iVICs was the difference in the extent of *ELOVL2* CpG islands methylation in the two cell types (Fig. 2). Although there was not a significant difference in the absolute amount of the C5 and C7 *ELOVL2* CpG methylation sites, we observed a more variable level of CpG methylation in iVICs and a more consistent and less variable epigenetic senescence in sVICs, with lower inter-individual variations. Whether differences in the general DNA methylation of the sVICs *vs*. iVICs reflects the presence of somatic mutations in epigenetically active enzymes such as DNMT3A and/or TET2, the determinants of the so-called clonal hematopoiesis of indeterminate potential (CHIP) strongly related to calcification of the aortic valve,^95^ is for now only a matter for speculation even though it may establish a rationale for an imbalance in the DNA methylation setting observed in the two classes of valve-resident cells.

### Senescence and calcification of human VICs is epigenetically established

The levels of histones post-translational modifications have been correlated to vascular aging and calcific disease. For example, it was found that an age-dependent increase in miR-34a determines the downregulation of SIRT1, a class IV HDAC,^46^ and this is crucially involved in vascular aging and calcification.^16^ However, the connection between histones post-translational modifications and valve calcific disease is less clear. In fact, from the relatively few available reports, it emerges that treatment of VICs with inhibitors of different HDAC classes may have beneficial, but also detrimental effects on VICs calcification.^88^ At the same time, in animal settings, the inhibition of p300 histone acetyltransferase was found to reduce VICs calcification by reducing the level of H3K9Ac in VICs derived from healthy valves.^89^

Because of these contrasting results, we decided to profile some histones post-translational modifications controlling active and repressed chromatin. By this approach, we found several differences in these marks, including higher levels of histones H3/H4 acetylation (H3K9Ac, H3K27Ac, and H4K16Ac), and a reciprocally inverted abundance of H3/H4 methylation (H3K27Met(3) and H4K20Me(3)) in iVICs *vs*. sVICs (Fig. 2). These results are at least in part conflicting with the existing literature produced using animal AoV-derived VICs,^87,88^ probably reflecting a different balance of chromatin remodelling enzymes and variations in the expression levels of the genes encoding for the various histone variants in the two human cell types compared with animal-derived VICs often employed in molecular studies.^96^ The existence of a clear difference in the expression of chromatin interacting/remodelling factors was confirmed by the analysis of specific transcripts presented in Fig. 2, whose results revealed variations in the expression level of several components of the PRC1/2 complexes,^34^ and DNA methylation readers connected with histone methylation/acetylation.^97^

### SPV106 inhibits the calcific evolution of stenotic VICs by reducing the senescent phenotype and modifying the epigenetic setup promoting histone acetylation

The increased cellular senescence in sVICs was reminiscent of what observed by Vecellio et al. in cardiac fibroblasts from diabetic *vs*. non-diabetic patients.^19^ In particular, in that study, it was found that the prolonged exposure to hyperglycaemia in vivo determined a senescent status of the cells determining a reduced ability to grow, an elevated DNA methylation, and a variation of crucial histone H3 marks, such as H3K9Ac (decreased), H3K9me(3) and H3K27met(3) (increased). Interestingly, in the present study, the reduced levels of histone acetylation coincided with a reduction in GCN5/pCAF histone acetyl-transferase activity, and treating cells with SPV106 reversed the senescent phenotype. In analogy with these findings, treating sVICs with the drug reduced, i) the level of senescence detected by the β-Gal staining in normal/high calcium-containing culture media, ii) the level of calcification, iii) the percentage of cells with nuclear p16, iv) the expression of p21 and, v) the percentage of cells expressing PCNA (Fig. 3). Importantly, the senescence-retarding effects of SPV106 lasted even after the drug withdrawal, suggesting, at least for the culture passages considered, the permanence of an epigenetic memory of the treatment in sVICs (Fig. 3). Conversely, treatment with garcinol, a HAT inhibitor, and ITSA, an HDAC activator, increased the senescence level of iVICs (Fig. 3). Altogether, these results show that altering the level of histone acetylation can accelerate or reduce senescence and consequent calcification in human VICs.

### Relevance of Notch pathway epigenetic control in reduction of VICs pathologic phenotype

Indications about possible epigenetically-controlled transcriptional pathways affected by SPV106 emerged from the analysis of transcripts differentially expressed in control *vs*. treated sVICs (Fig. 4). As expected, the pathways that were enriched with the highest significance described functions relative to chromatin modification/remodelling, and were characterized by a general downregulation of genes encoding for components of the chromatin modification and reading machinery. Given the absence of data on genomic regions that could be affected by different occupancy by some of these gene products (i.e., by ChIP-Seq), it is impossible to conclude what the transcriptional targets potentially interested in these changes are. On the other hand, it was interesting to note the implication of survival pathways including *PI3K* and *PTEN* genes and p53 activity involving acetylation^98^ and *TP53BP1*,^99^ and of pathways involved in SUMOylation and cellular senescence with concomitant upregulation of *BMI1* and *CDKN2A* (p16). This collectively suggests that treatment with SPV106 reduces cellular senescence and calcification by increasing cellular longevity,^54^ improving the repair of DNA double-strand breaks,^53^ or increasing self-renewal.^59,60^ A final important prediction emerging from the functional genomics analysis of the transcriptional signatures in SPV106-treated *vs*. control sVICs was the upregulation of pathways connected to Notch signalling, an essential effector of valve morphogenesis and disease.^20^ This prediction was validated by RT-qPCR using RNA extracted from cells treated with SPV106 in which an evident upregulation of *Notch1* and *Sox9* mRNAs and a decrease of *Runx2*, either at mRNA or protein/localization levels were observed. Furthermore, SPV106 enhanced nuclear transfer of the Notch intracellular domain with potentially wider transcriptional effects^64,100^ (Fig. 4). These results are in agreement with the transcriptional level of these genes observed in iVICs, in which expression of *Notch1* and *Sox9* was higher, and that of *Runx2* was lower than in sVICs, confirming the role of Notch in repression of VICs phenotype.^62^ In summary, activation of pCAF/KAT2B by SPV106 elicits a multilevel response in human VICs determining direct transcriptional effects on the control of master genes of VICs calcification under Notch-signalling control.

### Upregulation of Notch signalling by SPV106 occurs through genome-wide chromatin remodelling related to Histone acetylation

The transcriptional changes observed in sVICs treated with SPV106 (Fig. 4) were accompanied by an apparent reversion of histones H3/H4 acetylation and methylation marks to levels typical of iVICs (Figs. 2 and 5). Remarkably, these changes were neither associated to a reduction in general DNA methylation nor a specific demethylation of *ELOVL2* C2/C7 age-sensitive CpGs (Supplementary Fig. 10). Thus, even if SPV106 did not function as a canonical senolytic drug able to eliminate senescent cells from cultures and tissues^101^ and/or shifting back the epigenetic clock based on DNA methylation status,^92^ in our experiments it had a senomophic effect in retarding the sVICs senescence/calcification process by promoting a change in the transcriptionally active chromatin status related to H3K9Ac, H4K16Ac and H4K20me(3) histone marks.^102^ The genome-wide effects determined by the treatment on chromatin accessibility were confirmed by the ATACseq analysis performed on chromatin of DMSO- and SPV106-treated sVICs (Fig. 5). Apart from modifying chromatin accessibility in almost two thousand loci, the GO analysis of the genes with more open or more closed configurations, and thus bona fide more transcriptionally active or inactive, led to the identification of numerous pathways that were coherently regulated with the pathological status of the cells. For example, several networks encompassing genes with more open chromatin structure in SPV106-treated cells were related to the control of cell cycle/survival (e.g. by TP53 and PTEN) or, again, activation of the Notch-dependent pathway, confirming the transcriptional activation found by the transcriptomic profiling. By contrast, several networks encompassing genes with more closed chromatin configuration were related to Rho-signalling, a positive regulator of valve calcification.^103^ The experimental validation of ATACseq data by ChIP assays confirmed that the proximal promoter regions of *Notch1* and *Sox9* were associated with chromatin containing the activating H4K16Ac, but not the gene silencing H4K20Me(3), modification.^96^ This result is in line with previous evidences showing the relevance of the epigenetic control of *Notch1* for valve pathology,^104^ and clearly points to a direct engagement of the Histone H4 acetylation by pCAF/KAT2B activators in suppressing the senescence and the calcification of VICs *via* activation of NICD. At the moment, it is impossible to conclude on the functional significance of the other histone marks, e.g. the variation in the three-methylation and acetylation of H3K27, two histone modifications with antithetic function on Notch pathway regulation^105^ - detected by our analyses, as well as on the negative control of *Runx2* by SPV106 treatment. To address this will require specific experiments, such as targeted histone proteomics,^106^ and/or evaluation of complementary epigenetically-regulated pathways in control of valve calcification.^107^

### Histone acetylation maintains the structural integrity and protects the heart from valve disease-related functional deterioration

The ex vivo and in vivo results obtained, respectively, using a bioreactor-based and the Vitamin D-dependent calcification models^23^ (Fig. 6) shed light on the ability of the epigenetic treatment to reduce the deterioration of the valve due to hypercalcaemic conditions. In keeping with what reported for other anti-calcification treatments in genetic models of valve disease, e.g. the *Klotho* knockout mice,^108^ the employment of the drug reduced the occurrence of histologically-detectable calcifications and prevented the functional deterioration of the valve. Interestingly, in the ex vivo model, calcification in the valve leaflets was not characterized by abundance of cells with senescence markers such as p16 and p21 (Supplementary Fig. 15). This was expected, considering that the model has been designed to assess the effect of hemodynamic forces on valve calcification, likely occurring through endothelial to mesenchyme transition and not cellular senescence.^23^ On the other hand, even in this system, SPV106 determined a reduction of calcification and of cells expressing calcification markers, witnessing the role of epigenetics in control of VICs calcification, also independently of VICs senescent phenotype.

In vivo, the effect of SPV106 was demonstrated by an echocardiographic assessment of the heart function taking as a reference parameters describing the valve’s motion (the cusp separation), the transvalvular flow (time to peak)^78,79^ and the echocardiographic aortic valve scatter (Fig. 6). Interestingly, treatment with the epigenetic drug also protected the heart from the vitamin-D induced systolic dysfunction as assessed by the recovery of the ejection fraction. Although is not possible to exclude a direct cardioprotective effect of histone hyperacetylation, as observed in myocardial infarction models,^109^ we remark on the clinically exciting opportunity to maintain intact the systolic function by preserving the valve structural integrity.^110^

### Study limitations and concluding remarks

**Fig. 7:**
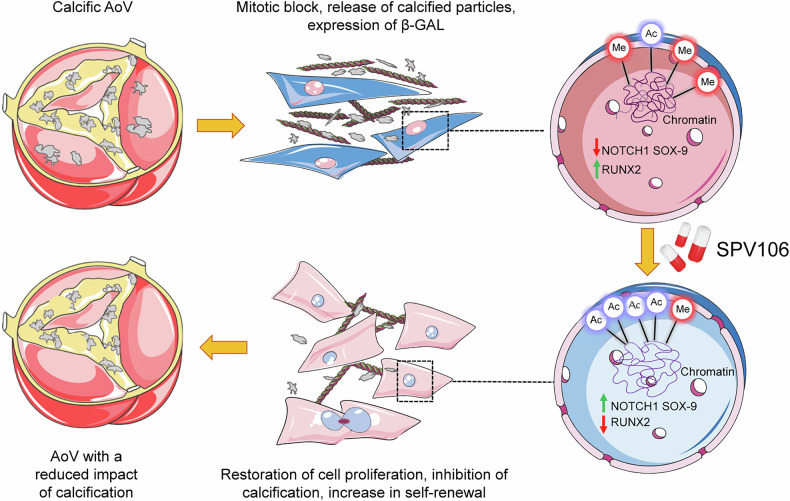
Proposed model of epigenetic-controlled senescence/calcification program of valve interstitial cells. The data presented in this study suggest that calcification of the aortic valve is associated to senescence of VICs due to decrease in histone acetylation and increase in histone methylation in chromatin. This situation can be reverted by SPV106, a drug with a genome-wide ability to restore histone acetylation, through upregulation of Notch-1 signalling and repression of osteogenic master genes (i.e. Runx-2). Other than inhibiting calcification, treatment with the drug also reduced the senescence level of the cells. This treatment could be amenable to establish treatment protocols to block/retard the progression of calcific disease in the human aortic valve based on KAT2B/pCAF activation. This image has been created with Servier Medical ART

To our knowledge, the present study is the first establishing a direct relationship between cellular senescence and the calcification potential of the human aortic VICs, linked by alterations in the histone epigenetic landscape (see model in Fig. 7). Compared to other studies performed with animal-derived cells, our work is realized with clinically-characterized human VICs. We recognize, however, that the lack of proper control cells to perform the cellular and molecular comparisons may represent a first limitation. Indeed, although they are biologically different from calcific valves cells, iVICs could still be affected by some predetermined pathologic programming resulting from modifications of the epigenetic setup compared to truly naive cells. In support of this is the evidence that even if treatment with SPV106 restored the histone marks found in iVICs, it did not reduce DNA methylation globally, nor at the level of the age-sensitive CpGs in the *ELOVL-2* gene promoter.

A second limitation of the study could be represented by our choice to exclude cells from the oldest donors, to match the chronological ages of the two-donor groups and exclude the chronological age as a confounding factor. While this might have caused an under-representation of the most extreme cell phenotypes, reducing the biological differences between the two groups, it helped us to delineate, for the first time and with a higher precision the epigenetic setup of the two pathologies and to find a possible treatment for aortic stenosis, the most prevalent of the two diseases and the one with a higher impact in elderly people.^111^ Given the lack of treatment specificity, direct valve targeting strategies (e.g. cell specific nanoparticle drug delivery) will have, however, to be adopted to avoid side effects of future treatments.

It is finally important to highlight that, while our results are coherent with the reported effect of the SPV106 on inhibition of the cellular senescence in diabetic cardiac fibroblasts and acceleration of cutaneous wounds closure,^19,112^ it also contrasts with numerous evidences showing that reduction of histone acetylation has a positive readout on VICs calcific programming.^87,89^ A second possibility to explain this contradiction is that the activation of KAT2B/pCAF might have a different readout on histone acetylation/methylation than that of treatment with histone deacetylase inhibitors employed in other reports, and that additional epigenetic modifications, in additions to those revealed in the present study, concur to reduce the senescent/calcific phenotype of sVICs treated with SPV106. More conclusive experiments using chromatin profiling techniques such as, e.g., ChiP-Seq or chromatin proteomics should be done to resolve this relevant issue. A third is that most of the HDACi studies have been performed with animal-derived cells, particularly porcine VICs.^87^ In this regard, a difference in the response of sVICs to treatment with SPV106 compared to HDACi could be contemplated. A final possibility is that the activation of KAT2B/pCAF by SPV106 has a direct effect on the expression or function of relevant genes involved in calcium homeostasis like Klotho or FGF23, as in part demonstrated in other cell types and in vivo in the literature.^113,114^ Studies are underway using reference models of valve calcific disease and naturally aged animals to discriminate between these possibilities.

## Materials and methods

### Human aortic valves collection and VICs isolation

The stenotic and insufficient aortic valves were collected during routine open chest aortic valve replacement procedures. The use of this material was approved by the ethical committee at Centro Cardiologico Monzino, IRCCS (first approval date June 19, 2012) and upon agreement on an informed consent. It also conformed to the Declaration of Helsinki. Primary human VICs (sVICs and iVICs) were isolated as previously described.^7^ The methods employed to perform specific analyses on cellular senescence and DNA/RNA material are specified in the supplementary information.

### Animal model of vascular/valve calcification by vitamin D administration

Animal procedures were performed in conformity with the guidelines from the Directive 2010/63/EU of the European Parliament on protecting animals used for scientific purposes and following experimental protocols approved by the Committee on Animal Resources of Cogentech (#824-2020). Male C57BL/6 J mice were used as already described.^14,16^ Further information about the model and the treatment of mice with SPV106, as well as the immunohistochemical and echocardiography methods that were employed to analyse the expression of markers and the valve function are epcficied in the online supplement.

### Ex-vivo model of aortic valve calcification

Experiments were performed with hearts of 3–6 months old mice (C57BL/6JRj) according to established protocols.^23,73^ The study was approved by the animal welfare committee of the Leiden University Medical Center and conformed with the guidelines from Directive 2010/63/EU of the European Parliament on the protection of animals used for scientific purposes. Details about the experimental procedure to induce valve calcification are provided in the online supplement.

### In vitro methods

Aortic valve interstitial cells were derived from pathologic aortic valves with insufficiency or stenosis. Cells were extracted following a simple digestion method as already described by us.^7^ Evaluation of cellular senescence was performed according to beta-Gal staining and doubling time while other parameters like expression of senescence markers, calcification, epigenetic status, chromatin accessibility, RNA profiling and bioinformatics are specified in the online supplement.

### Statistical analysis

Data analyses and graphing were performed using GraphPad Prism software (version 9.1.2). The general criteria adopted in the cell biology data analyses was to use unpaired t-test/ANOVA when comparing cells from different donors (e.g., typically the iVICs *vs*. sVICs comparisons), and to employ pairwise methods to assess the effects of treatments on cells (e.g., the effects of drugs on sVICs *vs*. DMSO-treated sVICs). The circles overlapped to each plot and bar graphs represent the individual values of independent samples (cells from different donors) and their sum indicates the *n* of the samples included in the analyses.

## Supplementary information

online supplement

Dataset containing ATAC-seq raw data

## Data Availability

The quantitative data supporting this article are publicly available at Zenodo data repository. The corresponding DOIs are as it follows: 10.5281/zenodo.16895587; 10.5281/zenodo.16874826; 10.5281/zenodo.16872541; 10.5281/zenodo.16874842; 10.5281/zenodo.16900932.
