# Supplementary material for: Reversion of aortic valve cells calcification by activation of Notch signalling via histone acetylation induction
Source: Signal Transduct Target Ther. 2025 Sep 18;10:311. doi: 10.1038/s41392-025-02411-8 (PMC12443999; doi:10.1038/s41392-025-02411-8)
Supplement: Supplementary file 1 — online supplement [file 41392_2025_2411_MOESM1_ESM.docx]

Supplementary Materials for

**Reversion of aortic valve cells calcification by activation of Notch signalling *via* histone acetylation induction**

Gloria Garoffolo^1^, Silvia Ferrari^1^, Sara De Martino^2^, Emanuele Pizzo^1^, Veronica Candino^1^, Lavinia Curini^1^, Federica Macrì^1^, Boudewijn P.T. Kruithof^3,4^, Alessia Mongelli^5,6^, Magda Grillo^1^, Nadia Fanotti^1^, Pamela Fejzaj^1^, Manuel Casaburo^1^, Azizah Alanazi^7^, Nina Ajmone Marsan^3^, Feras Khaliel^7^, Ahmed Alsulbud^7^, Marco Agrifoglio^1,8^, Gualtiero I. Colombo^1^, Mattia Chiesa^1^, Antonella Farsetti^2^, Carlo Gaetano^5^, Angela Raucci^1^, and Maurizio Pesce^1, 7, 9, 10, *^

Correspondence to [maurizio.pesce@ccfm.it](mailto:maurizio.pesce@ccfm.it); [mpesce@kfshrc.edu.sa](mailto:mpesce@kfshrc.edu.sa)

**This PDF file includes:**

Materials and Methods

Figures S1 to S16

Tables S1 to S7

Caption for Data S1

**Other Supplementary Materials for this manuscript include the following:**

Supplementary references

**Materials and methods**

**Animal model of vascular/valve calcification by vitamin-D administration**

Male C57BL/6J mice were purchased from Charles River Laboratories (Calco, Italy). Nine weeks old mice were treated with either 500.000 IU/kg/day vitamin D (Cholecalciferolor, C1357, Sigma-Aldrich, St. Louis, MO, USA) or a mock solution (1% (v/v) ethanol, 7% (v/v) Kolliphor® EL and 3.75% (w/v), dextrose (Sigma-Aldrich, St. Louis, MO) administrated subcutaneously for three consecutive days and sacrificed seven days after the first injection as already described in ^1,2^. Animals were anesthetized with an intraperitoneal injection of ketamine (100 mg/Kg) and perfused with PBS. Organs were processed as described below. Aortic valve was fixed in 10% formalin and paraffin embedded. Seven μm sections were de-paraffinized and incubated with 1% silver nitrate solution (Sigma-Aldrich, St. Louis, MO) under ultraviolet light for 50 minutes. After rinsing the specimens with several changes of distilled water, the unreacted silver was removed with 5% sodium thiosulfate for 5 minutes (Sigma-Aldrich, St. Louis, MO) at room temperature. Then, the sections were rinsed in distilled water and counterstained with hematossilin and eosin ^2^. Aortic valve section images were taken with an Axioskop II microscope (Zeiss, Oberkochen, Germany) equipped with a digital camera (AxioCam Color, Zeiss). The Von Kossa positive area was analyzed with Axiovision Software Rel 4.7 (Zeiss) and the positive percentage was defined as the fraction of Von Kossa positive staining to the total area (µm^2^). Aortic valve was fixed in 10% formalin and paraffin embedded. Seven μm sections were boiled for 20 min in Dako Target Retrieval Solution Citrate pH 9 (Agilent Technologies, Santa Clara, CA, USA) and then blocked with 5% goat serum (Sigma-Aldrich, St. Louis, MO) in 0.1% PBS-Triton X-100 (PBS-T, Sigma-Aldrich, St. Louis, MO) for 60 min at room temperature. Primary antibody against p21 (12 ng/mL, #ab109199, Abcam, Cambridge, United Kingdom) and Osteopontin (10 ng/ml, #ab63856, Abcam, Cambridge, United Kingdom) were dissolved in 1% goat serum PBS-T and incubated overnight at 4 ◦C in a humidified chamber. Sections were counterstained with hematoxylin. Images were acquired with an Axioskop II microscope (Zeiss) equipped with a digital camera (AxioCam Color, Zeiss).

**In vivo cardiac and aortic valve function**

Transthoracic echocardiography was performed under ~2% isoflurane anesthesia. The images were acquired by Vevo 3100 Imaging system (Visualsonics, Toronto, Canada) equipped with a 25-55 MHz (MX550D) transducer. By this approach, parasternal short axis view of the left ventricle (LV) was employed to evaluate the LV ejection fraction (EF) and Heart Rate (HR). Aortic cuspid separation (ACS) was assessed in the left parasternal long-axis view using M-mode imaging at the level of the aortic valve. The measurement was taken as the distance between the posterior aortic wall and the inner edge of the aortic valve to evaluate the extent of valve opening or closure. The average of at least two consecutive cardiac cycles was used for each measurement. Aortic valve time to peak (Tp) velocity was measured in the suprasternal view with a pulse-wave-Doppler using angle correction of 45°. The Tp was measured as the time from the opening of the aortic valve to the point when the flow velocity across it reaches its peak. The average of three consecutive cardiac cycles was used for each measurement. Two-dimensional ultrasonic echocardiography back scatter AVBS imaging of the aortic valves was performed as previously described using a standard parasternal long-axis-like view, capturing data over three cardiac cycles^3^. Regions of interest were selected on valve leaflets using 7 × 7-pixel sample volumes. Backscatter measurements from the blood pool near the left ventricular outflow tract and the aortic blood pool served as reference points to obtain AVBS values. Because heart rate of>400 beats/min is advised to be within the physiological range of murine heart rate under anesthesia, recordings in which heart rate was >400 beats/min were used for analysis of data obtained in anesthetized mice.

**Ex vivo mouse aortic valve calcification model**

Before collection of the hearts for ex vivo culturing, mice were anesthetized with an intraperitoneal injection of ketamine (100 mg/Kg) and xylazine (10 mg/kg), followed an intraperitoneal injection of 100 μl of heparin (5000 IU/ml). The hearts were in situ perfused with salt solutions, isolated and cultured in the Miniature Tissue Culture System (MTCS) for 7 days as previously described ^4,5^. Flow (1000 µl/min) was introduced using a pump and medium (DMEM; Life technologies, 31966047, supplemented with 10% fetal bovine serum, antibiotic/antimycotic (A/A; 100 units of penicillin,100 μg of streptomycin, and 0.25 ug of amphotericin B/ml), Insulin-Transferrin-Selenium (ITS; 10 μg/ml insulin, 5.5 μg/ml Transferrin, 6.7 ng/ml Selenium) supplemented with 3 mM sodium phosphate (Sigma) and with or without 15 uM SPV106 (SML0154; Sigma) was perfused through the aorta towards the closed aortic valve into the coronary circulation. The medium was replaced after 3 days. The hearts were fixed overnight with 4% PFA/PBS and histology was performed as previously described1. Alizarin red staining was performed for visualization of the calcification. For immunofluorescence primary antibodies directed against RUNX1 + RUNX3 + RUNX2 (RUNX1/2/3; Abcam; [EPR3099] ab92336; 1:100), ALP (R&D; AF2910; 1:250) and alexa-conjugated secondary antibodies (Molecular Probes) were used. Slides were mounted using DAPI containing ProlongGold Antifade reagent (Thermofisher), scanned with the Pannoramic 250 slide scanner (version1.23, 3DHISTECH Ltd.) and analyzed using Caseviewer (version2.3, 3DHISTECH Ltd.). The calcification in the aortic valve in each heart was quantified by taking the average of the alizarin red-positive area of at least 6 sections with 96 µm interval. To quantify the RUNX1/2/3 expression in the aortic valve, the percentage of RUNX1/2/3-positive cells of the aortic valve was determined, whereas the ALP-expression was quantified by determining the ALP-positive area.

**Human VICs isolation**

For the isolation of human valve interstitial cells, a brief preliminary leaflet incubation of the removed valve cusps in Collagenase Type II solution (1000 U/mL, Worthington) was performed under gentle shaking at 37°C to remove the endothelial cell layer. The time of incubation (5 minutes) was identical for insufficient and stenotic valves. VICs were isolated by a second incubation, under the same conditions, for two hours. Cells were plated for in vitro amplification on 1% gelatin-coated plastic cell culture dishes and cultured in DMEM (Lonza) supplemented with 150 U/mL penicillin/streptomycin (Sigma Aldrich), 2mM L-glutamine (Sigma Aldrich), and 10% bovine serum (HyClone, Thermo Scientific). Experiments with SPV106 (Sigma Aldrich) were performed on sVICs at passage 5 of culture, 30% confluence, in the presence or absence of the drug. The drug was dissolved in DMSO and added to the medium at different final concentrations (5, 15, 25 μM) for seven days before β-gal staining. An equal concentration of DMSO was added to the medium used for control cells. To test the effects of SPV106 on sVICs on calcification potential, cells were treated with SPV106 and grown in a standard or pro-calcific medium. SPV106 was substituted every two days. Experiments with Garcinol, an inhibitor of p300 and other Histone acetylases (HATs), were performed with iVICs, again at passage 5 of culture^6^.

**Global DNA methylation assay**

Global levels of 5mC, 5hmC were measured by the MethylFlash Methylated DNA Quantification Kit (Epigentek), MethylFlash Hydroxymethylated DNA Quantification Kit (Epigentek). According to the manufacturer's instructions, genomic DNA was extracted from VICs (at the 3^rd^ passage) using DNeasy Blood & Tissue Kit (Qiagen). 100 ng per reaction was used to detect 5mC, and 5hmC levels using capture and detection antibodies and then quantified colorimetrically by reading the absorbance in a microplate spectrophotometer (TECAN Infinite M200 PRO).

**Acidic β-galactosidase assay**

Acidic β-Galactosidase Assay Senescence-associated acidic -galactosidase was assayed following the manufacturer's instructions (Cell Signaling Technology) at the indicated passage numbers (**Figure 2 c, d**). Briefly, cells were fixed with a fixative solution (Cell-Signaling) for 10-15 min at room temperature (RT) and subsequently incubated with a specific staining solution (Cell-Signaling - final pH of 6.0) at 37°C, overnight, in a dry incubator (without CO2, which may change the pH, possibly affecting final results). Stained cells were then observed under a light microscope, at 20X magnification, and images were analyzed by ImageJ software.

**In-cell western assay**

Cells at passage 3 (or 5 for SPV106 treatment) were plated at 70% confluence overnight in 96 multi-well plates, fixed in 4% paraformaldehyde (10 mins, RT), permeabilized with 0.1% Triton X-100 (25 min; RT), and then blocked with 3% BSA/PBS (1 h, RT). Cells were first incubated with different primary antibodies (H3K9Ac, H3K27Ac, H3K27me3, H4K16Ac, H4K20me3; 3.54 μg/mL – all from AbCam) at 4°C, overnight, under agitation, and subsequently with secondary antibody IRDyes 700/800 (LI-COR) (1 h, RT). According to datasheet instructions, signals were normalized for total DNA content by DRAQ5 (20 μg/mL - AbCam) counterstaining. The Odyssey Infrared Imaging System (LI-COR Biosciences) performed detection, and images were analyzed with Image J software.

**Western Blot and ELISA**

For Western blotting analyses, cells were lysed in a buffer containing 10 mM Tris-Cl, pH 7.4, 150 mM NaCl, 5 mM EDTA, 1% Triton X-100, 1% sodium deoxycholate, 0.1%, sodium dodecyl sulfate and 1%, protease and phosphatase inhibitor mixture (Sigma-Aldrich). Whole cell lysates were sonicated, centrifuged for 15 min at 14 000 g; cell supernatants were then collected and proteins were quantified by BCA protein assay kit (Pierce Chemical Co). Cell lysates (30μg) were diluted in Laemli sample buffer, heated at 95 °C for 5 minutes, run onto 4-12% gradient SDS polyacrylamide gels (Invitrogen), and transferred to nitrocellulose membranes. The blots were then blocked with Tris Buffered-saline containing 5% BSA at RT for 1 hour. Overnight incubation at 4 °C with anti- p21, anti-RUNX-2 and GAPDH primary antibodies (diluted as indicated, Cell Signalling) was performed, followed by incubation with appropriate secondary antibodies for 20 minutes. Images were acquired with ChemiDoc and band intensities were quantified using ImageJ software. An enzyme-linked immunosorbent assay (ELISA, ARG80929, Arigo biolaboratories) was performed on conditioned medium of SPV106 treated cells vs.control according to the manufacturer's instructions to detect the levels of human inflammatory cytokines. Calibration curves were prepared using purified standards for the protein assessed and curve fitting was accomplished by regression following the manufacturer's instructions.

**Immunofluorescence**

Immunofluorescence (IF) analyses on cells at passage 5 (sVICs, iVICs or sVICs ± SPV106) was performed after fixing with paraformaldehyde 4% (10 min, RT). First, cells were incubated in a blocking solution consisting of PBS containing Triton-X 100 (1%), BSA 3% (1h, RT) and then incubated with Anti-CDKN2A/p16INK4a (3 μg/mL, AbCam), anti PCNA (Dako, 3.27 μg/mL), anti-NICD antibody (2 μg/mL, Santa Cruz Biotechnology) or anti-acetyl lysine antibody (2 μg/mL, Abcam) or anti-SOX9 antibody (12,3 μg/mL, Abcam), overnight at 4°C. Subsequently, cells were incubated with secondary anti mouse/rabbit –alexa594 antibody or secondary anti mouse-alexa488 antibody [1 h, RT (Invitrogen)]. F-actin cytoskeleton was stained using Phalloidin (Sigma Aldrich, 32 μg/mL), and nuclei were counterstained with DAPI (DAKO, 50 μg/mL). Image analysis of NICD nuclear fluorescence was performed as described by us recently^7^. Imaging of the cells was performed with a Zeiss LSM710 confocal microscope. Fluorescence quantification was made using Image-J software as described^7^ on images shot with the same laser intensity and photo-multiplication.

***In vitro* cell calcification**

Cells at passage 3 were seeded at 70% confluence in a 96 multi-well plate. After overnight incubation, cells were treated with a calcification medium (cultured medium supplemented with 2 mM Na2HPO4) or standard medium. Normal culture medium or pro-calcific medium was changed every two days for 1 week. To quantify calcification, after 7 days of treatment, we added 200 μl of HCl 0.6 M to each well, left it for 5/6 hours at 4 °C under agitation, and analyzed it with Calcium Colorimetric Assay Kit (BioVision Incorporated) according to the manufacturer. The total calcium amount was normalized to the total protein content in the cell lysates. The preliminary testing of this assay showing the differences in intracellular calcium accumulation in sVICs cultured under normal or pro-calcifying conditions is shown in **Supplementary Fig 1**. For Von Kossa staining, cells cultured in pro-calcific medium (± SPV106) were fixed with paraformaldehyde 4% (10 min, RT) and incubated with 1% silver nitrate solution under UV for 30 min. After rinsing the wells with several changes of distilled H2O, the unreacted silver was removed with 5% sodium thiosulfate for 5 min at room temperature. Images were taken using Axiovert 40 C microscope (Zeiss, Oberkochen, Germany). Quantification of the Von Kossa-stained area as a percentage of the total area was carried out using ImageJ software. For Alizarin staining, sVICs at passage 3 (95% confluence), were incubated in twenty four-well plates in osteogenic medium consisting of Dulbecco’s modified Eagle’s medium (DMEM) with 4.5gL^-1^ glucose, 10 % FBS, 100 U/ml penicillin, 0.1 mg/ml streptomycin, 2 mM inorganic phosphate Na2HPo4 (PH:7.4), and 100ng Lipopolysaccharide (LPS). The medium was replaced every 2 days for 7 days. Mineralization was detected after cell fixation with 4% Paraformaldehyde (15 min), and Alizarin Red S staining (50 min) to evaluate calcium deposits. Cells were rinsed two or three times with PBS to reduce non-specific staining to terminate staining before microscopic evaluation.

**MTT assay**

To evaluate the cellular vitality, sVICs treated or not with SPV106 (15 Μm) were incubated with 3-(4,5-dimethylthiazol-2-yl)-2,5-diphenyltetrazolium bromide (MTT) solution (5 mg/ml) for 4 hours at 37°C. For MTT quantification, the staining was solubilized with DMSO and optical density (540 nm) was measured using Infinite M200 PRO reader (Tecan).

**DNA pyrosequencing**

1.5 10^6^ cells at passage 3 were trypsinized and suspended in 180 µL of ATL buffer (Qiagen) and 20 µl of proteinase K (Qiagen), and incubated for 10 minutes at 56°C. Then an automatic DNA extraction was performed by QiaCube (Qiagen) according to manufacturer instructions following the protocol QIAmp DNA mini. Then, 2 µL DNA was quantified with QIAxpert (Qiagen, cat. 9002340). Next, 500 ng of DNA has been used to convert with Epitect fast DNA bisulfite (Qiagen) following the manufacturer instructions associated with RotorGene 2plex HRM (Qiagen), which performs the denaturation and incubation steps (95°C for 5 minutes and 60°C for 10 minutes respectively) for two times. The QIACube automated system was finally used to purify the converted DNA. Finally, 2µL of converted DNA has been quantified with QIAxpert. Successively, PCR reaction mixes were performed using PyroMark PCR kit (Qiagen) using 60 ng of bisulfite converted DNA using specific primers for the *ELOVL2* methylation marks^8^ by RotorGene 2plex HRM (Qiagen). Finally, to detect the methylation level of *ELOVL2* CpG sites, PCR amplicons were sequenced with the PyroMark Q24 system (Qiagen) according to the manufacturer's, using 5µl of PCR product and 20µl of sequencing primer at the concentration of 0.375 µM ^8^.

**Statistical/bioinformatic analysis of ATAC Seq and RNA array data**

The raw data of ATAC-seq and transcriptomics have been deposited at Zenodo public repository with the following accession numbers: Array chromatin remodelling (**Table S2**) - 10.5281/zenodo.16872541; Array senescence (**Table S3**) - 10.5281/zenodo.16874826; ATAC-seq - 10.5281/zenodo.16874842 (**Data S1**). Analysis of ATAC-seq data was performed by aligning the reads with the BWA algorithm (mem mode; default settings). Duplicate reads were removed, only reads mapping as matched pairs and only uniquely mapped reads (mapping quality ≥ 1) were used for further analysis. Alignments were extended *in silico* at their 3’-ends to a length of 200 bp and assigned to 32-nt bins along the genome. The resulting histograms (genomic “signal maps”) were stored in bigWig files. Peaks were identified using the MACS 2.1.0 algorithm at a cutoff of p-value 1e-7, without control file, and with the –nomodel option. Peaks that were on the ENCODE blacklist of known false ChIP-Seq peaks were removed. Signal maps and peak locations were used as input data to Active Motifs proprietary analysis program, which creates Excel tables containing detailed information on sample comparison, peak metrics, peak locations and gene annotations. The ‘DaMiRseq’ R package^9^ was used to filter out inaccessible loci (less than 20 counts in more than 90% of samples), perform the data normalization (variance stabilizing transformation), and the exploratory analysis. The differential analysis (paired test) was performed by the ‘limma’ R package^10^. A locus was deemed significant whether the *P*-value was < 0.05. The pathways analysis was performed using the Cytoscape^11^ (v. 3.9.1) plug-in ClueGO^12^ (v. 2.5.8), which estimates the pathways enrichment score on pre-selected set of genes, exploiting a two-sided hypergeometric test. The Reactome database^13^ has been selected as reference. Pathways with less than 4 associated genes from the uploaded gene list were discharged. Functional related Reactome terms were grouped by setting a similarity threshold of kappa score of 0.4. Pathways with an associated *P*-value < 0.05 were deemed as significant.

Data collected with RT^2^ Profiler PCR Arrays were expressed as normalized relative expression levels, calculated with the 2^-ΔΔCt^ method. The calculation was done as follows: ***i)*** to obtain an “average” reference gene for each experiment, we computed the geometric mean of the Ct of the 5 reference genes included in the arrays (*ACTB*, *B2M*, *GAPDH*, *HPRT1*, and *RPLP0*), for each conditions and biological replicate; ***ii)*** to obtain an “average” calibrator for each gene, we computed the geometric mean of the Ct of each target gene in the control conditions; **iii)** we therefore computed the ΔCt of the calibrator (Ct_calibrator_ – Ct_reference gene_) and of each gene of interest (Ct_target gene_ – Ct_reference gene_) for each condition and biological replicate; ***iv)*** finally, we calculated the 2^-ΔΔCt^ (ΔCt_target gene_ – ΔCt_calibrator_) value. Normalized expression values were log_2_-transformed and differences in gene expression were determined by unpaired or paired *t* test, when appropriate, assuming an individual variance for each gene. To control for the false discovery rate (FDR), *q*-values were estimated using the two-stage step-up method of Benjamini, Krieger, and Yekutieli. An FDR adjusted *P*-value (*q*-value) < 0.1 was deemed as significant. Hierarchical clustering was performed to visualize the differences in gene expression between each group. Normalized expression values were mean-centered and genes and samples were clustered by Pearson’s correlation, as distance metric, and the average linkage method with order optimization. Clustering analyses were performed using the open-source software MultiExperiment Viewer (MeV) version 4.9. The Cytoscape (version 3.8.2) plug-in ClueGO (version 2.5.8) was used to perform functional enrichment analysis, again using the Reactome Pathway Database as ontology source, and visualize the non-redundant biological terms for the cluster of differentially expressed genes in a functionally grouped network. Enrichment analysis was performed using the 2-sided hypergeometric test and terms were considered significant for a Bonferroni corrected *P*-value < 0.05.

**RNA analyses**

Total RNA was extracted from cells at passage 3 (or 5 for SPV106 treatment) using the Trizol reagent (Thermo Fisher Scientific) and a column-based protocol (Zymo Research), including a DNAse treatment. RNA was reverse-transcribed using RT2 First Strand Kit (Qiagen) under the following reaction conditions: 42°C for 5 min, 4° C for 1 min, 42°C for 15 min and 95°C for 5 minutes. The cDNA obtained was used to perform real-time RT2 Profiler PCR Arrays (Qiagen) in combination with RT2 SYBR Green qPCR Mastermix (Qiagen). Each of the PCR array plates contained primers set for 96 genes associated with human Cellular Senescence, Chromatin Modification, and Chromatin Remodeling Factors (Qiagen catalog numbers PAHS-050Z, PAHS-085Z and PAHS-086Z, respectively). The same procedure was followed to perform Quantitative realtime PCR (qRT-PCR) to detect *Notch1*, *Sox9*, *Runx2* and *OPN* gene transcripts (primers details in **Table S5**), using Power SYBER Green PCR Master Mix (Applied Biosystems) on a 7900 Fast Real-Time PCR System (Applied Biosystems). Finally, gene expression levels were normalized to GAPDH expression data and statistical analyses were performed on ∆Ct values.

**Chromatin immunoprecipitation (ChIP)**

ChIPs were performed using Magna ChIP™ A/G Chromatin Immunoprecipitation Kit (Sigma-Aldrich, #17-10085), according to manufacturer’s instructions. The preamplification step was performed using 4 μl of DNA with SYBR Master Mix (Applied Biosystems, Foster City, CA, USA) and primer mix at 50nM final concentration (PCR: 14 cycles at 95°C for 15 s and at 58°C for 4 min). Analysis of DNA fragments was performed by qPCR on QuantStudio 5 Real-Time PCR System (Applied Biosystems, Foster City, CA, USA) using SYBR Master Mix (Applied Biosystems, Foster City, CA, USA) with the evaluation of dissociation curves. Standard curves were generated by serially diluting DNA input and data normalized to corresponding inputs were expressed as relative enrichment^14^. Immunoprecipitations were performed using specific antibodies to H4K16ac (Active Motif #39929, Carlsbad, CA, USA) and H4K20me3 (Abcam, #ab9053). IgG (Bethyl. #P120-101, Montgomery, TX, USA) was used as negative control. Primer sequences (forward and reverse, respectively) are listed below:

hNOTCH1prom (-1000 bp from TSS) 5’- GCACGAGGCCGTGAACTTT -3’ and 5’- CCTGTGCCAAGCCTGGTTAA -3’.

hSOX9prom (-1000 bp from TSS) 5’- AGCAGGCAAGCAGCATGACT -3’ and 5’- GGCACCACCGCAGACAA -3’.

hRUNX2prom (-1000 bp from TSS) 5’- AGAATGCCCAGACTAACACTTTCA -3’ and 5’- AAGGTCTGGTTGCAAACTGCTT -3’.

hNOTCH1prom (-1500 bp from TSS) 5’- GCCCCGCGCTTCCTT -3’ and 5’- GGCAGCAGAGATGTTTATGTAACAG -3’.

hNOTCH1prom (+0 bp, TSS) 5’- TGCAAATTTCAGTCGCCAGTT -3’ and 5’- TTTTCAGAGGCCAAAAGTTTGAG -3’.

**
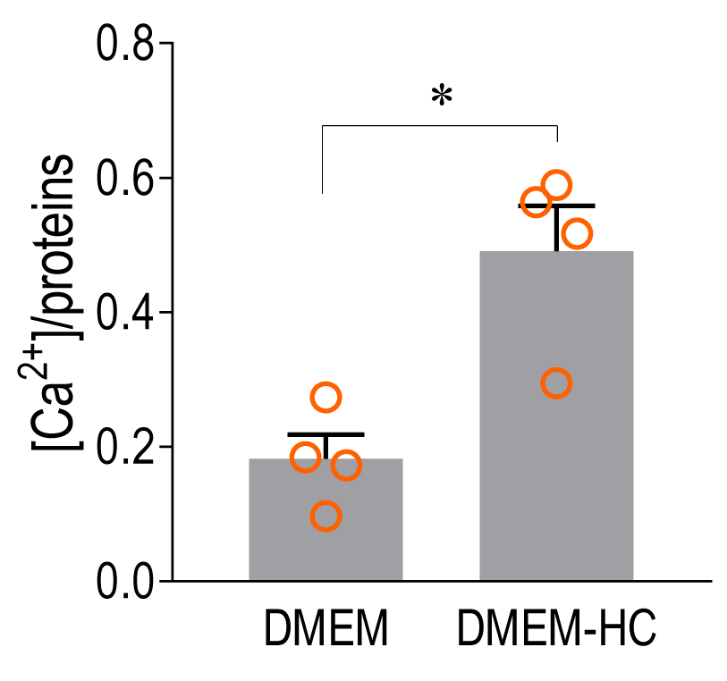
**

**Supplementary Fig 1.** Setting of the *in vitro* calcification assay as described in the methodological section. The bar graph shows the level of intracellular calcium normalized over the total amount of proteins accumulated by the cells when they were plated in pro-calcific conditions in DMEM containing high calcium (HC) level. Above bar graph **P* < 0.05 by paired t-test. The *n* of biological replicates is 4.

**
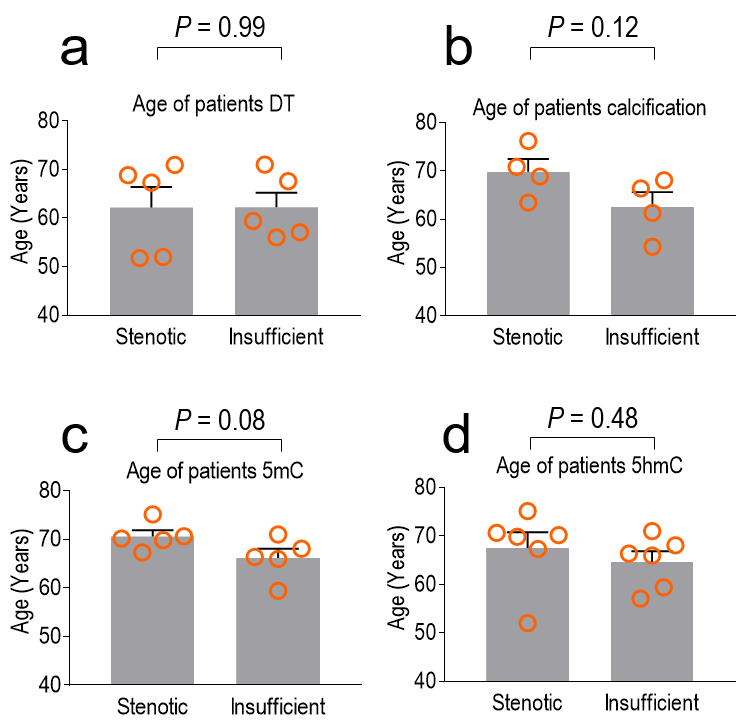
**

**Supplementary Fig 2.** Comparison between the chronological age of iVICs and sVICs donors used for experiments in **Figure 2** for determination of the doubling time, β-Galactosidase expression calcification potential; and 5mC and 5hmC DNA content, respectively. The *P* values of the statistical analyses (unpaired t-test) performed to exclude significant differences in the age of cell donors included in the analyses are indicated above each plot. The *n* of biological replicates is represented by the number of dots overlapped to each of the histogram plots, each indicating an individual cell donor

**
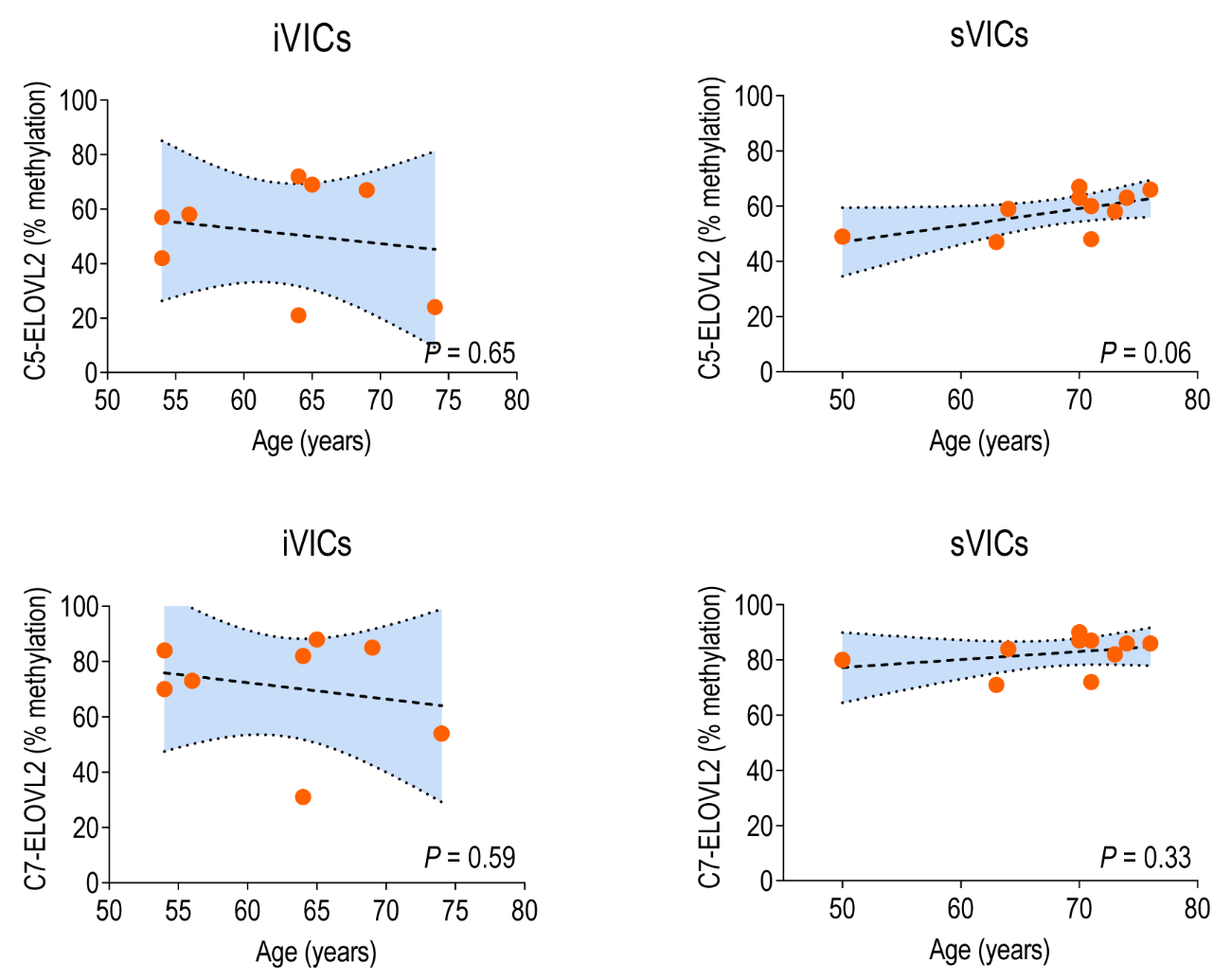
**

**Supplementary Fig 3.** Lack of correlation between chronological age of the iVICs and sVICs donors and methylation level (expressed as a percentage of total, as indicated in **Figure 2**) of the biological age-sensitive CpG islands 5 and 7 in the *ELOVL2* gene promoter. Plots show the results of regression analysis of the methylation level against the chronological age of each cell donor for both methylation hotspots. In each plot, the areas in color indicate the 95% confidence intervals of the best-fit data interpolation (dashed line). The *n* of biological replicates is 8 in graphs representing iVICs and 10 in graphs representing sVICs.

**
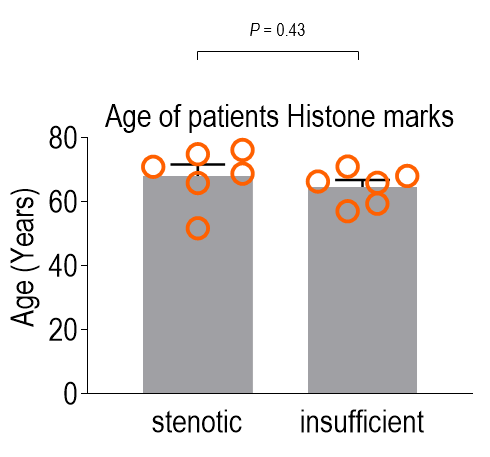
**

**Supplementary Fig 4.** Comparison between the chronological age of iVICs and sVICs donors used for experiments for determination of the levels of the 5 Histones H3/H4 marks in **Figure 2.** The P value of the statistical analyses (unpaired t-test) performed to exclude significant differences in the chronological ages of cell donors is indicated above the plot. The n of biological replicates is represented by the number of dots overlapped to the histogram, indicating an individual cell donor.

**
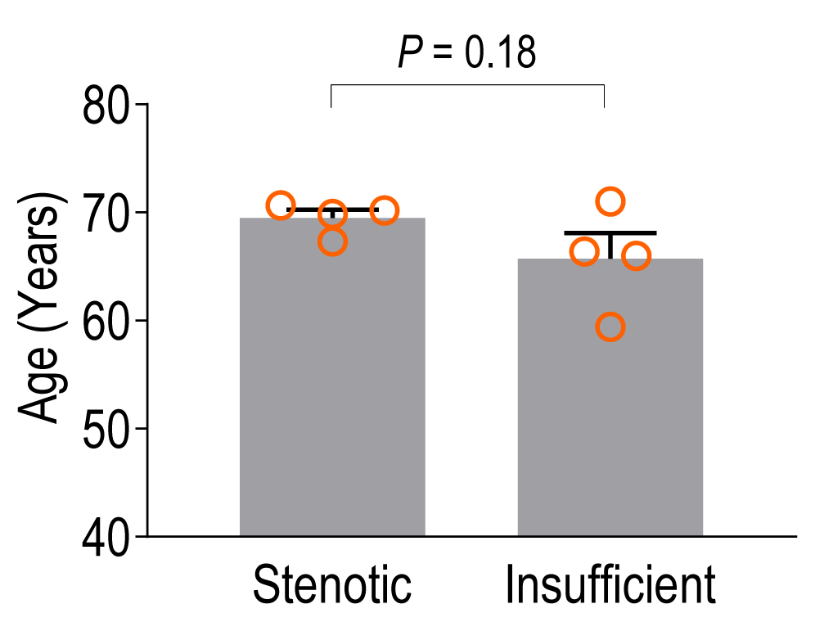
**

**Supplementary Fig 5.** Comparison between the age of iVICs and sVICs donors used for experiments for mRNA analyses described in **Figure 2**. The n of biological replicates is represented by the number of dots overlapped to each of the histogram plots, each indicating an individual cell donor

**
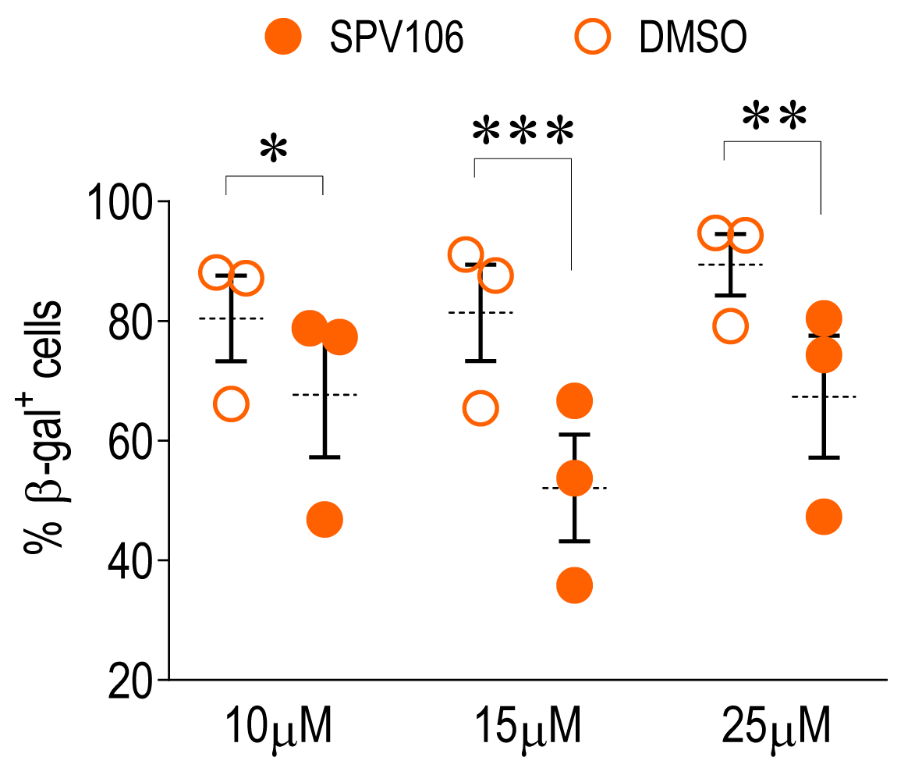
**

**Supplementary Fig 6.** Assessment of the best SPV106 concentration to reduce cell senescence in sVICs. The plot shows the percentages of the β-Gal^+^ cells in the presence of three SPV106 concentrations. According to the two-ways ANOVA (repeated measures), 15µM was the concentration that produced the best difference between control sVICs and sVICs treated with SPV106. The *P* values of the 2-way ANOVA Bonferroni post-hoc test that was used to assess the significance of the comparisons between the different concentrations *vs.* their relative controls, are indicated by asterisks as it follows: * *P* < 0.05; ** *P* < 0.01; *** *P* < 0.001. The *n* of biological replicates is 3.

**Supplementary Fig 7.** Staining with MTT does not reveal a negative effect of SPV106 treatment on sVICs vitality. The n of biological replicates is represented by the number of dots overlapped to each of the histogram plots, each indicating an individual cell donor.


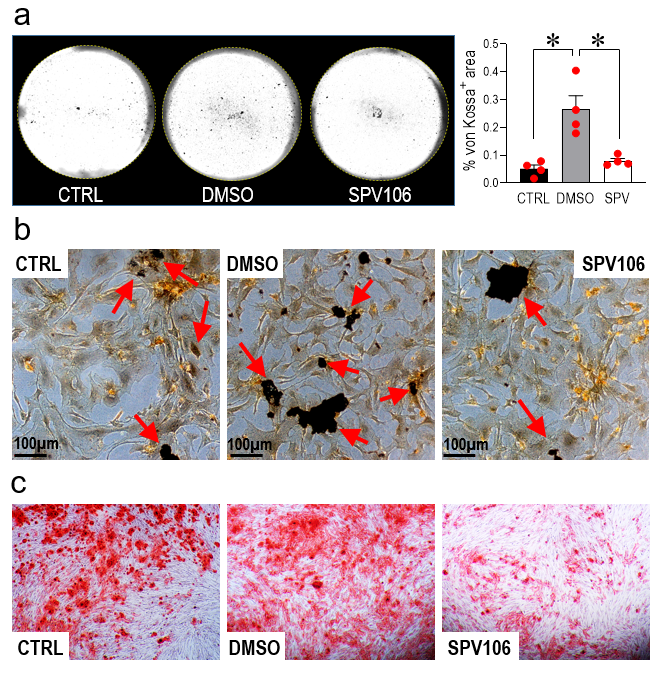


**Supplementary Fig 8. Calcification of sVICs exposed to osteogenic medium is inhibited by SPV 106 treatment.** Panels a) and b) show low and high magnifications of Von Kossa staining of CTRL, DMSO- and SVP106-treated sVICs. As evident by the micrographs and the quantification, treatment with SPV106 reduced at calcification at the levels of cells cultured in low calcium medium. A similar decrease of calcification was observed also by staining cells with Alizarin Red (panel c), confirming the anti-calcification effect of SPV106 on human VICs, in addition to the anti-senescence effect reported in **Figure 3.**

**
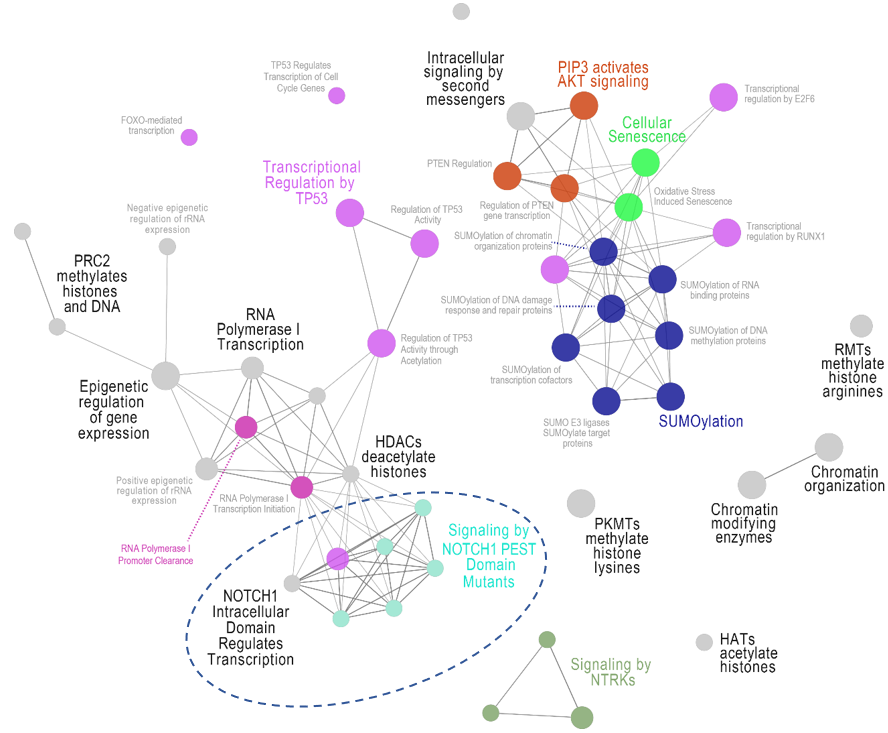
**

**Supplementary Fig 9.** ClueGo network of Reactome pathways enriched in the SPV106-modulated genes grouped by significant terms (in bolds; pathways listed in **Table S4**). It is evident the enrichment of pathways with a functional annotation relative to chromatin remodelling, cellular senescence, PI3K/AKT signalling, SUMOylation and Notch1 (encircled).

**
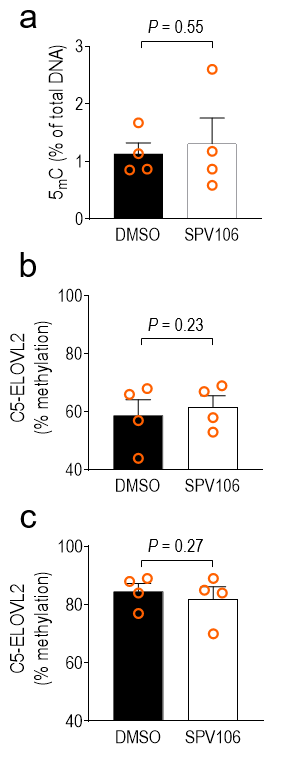
**

**Supplementary Fig 10.** SPV106 does not alter DNA methylation levels neither at a global level nor at the ELOVL2 gene promoter CpG islands 5 and 7. (**a**) The plot shows the results of the global DNA methylation in control cells and cells treated with SPV106, as detected by ELISA test. (**b**, **c**) show the methylation level of the C5 and C7 ELOVL2 CpG islands, respectively, as detected by bisulfite conversion followed by pyrosequencing. The *P* values of the statistical comparison by paired t-test are shown above each plot. The *n* of biological replicates is represented by the number of dots overlapped to each of the histogram plots, each indicating an individual cell donor


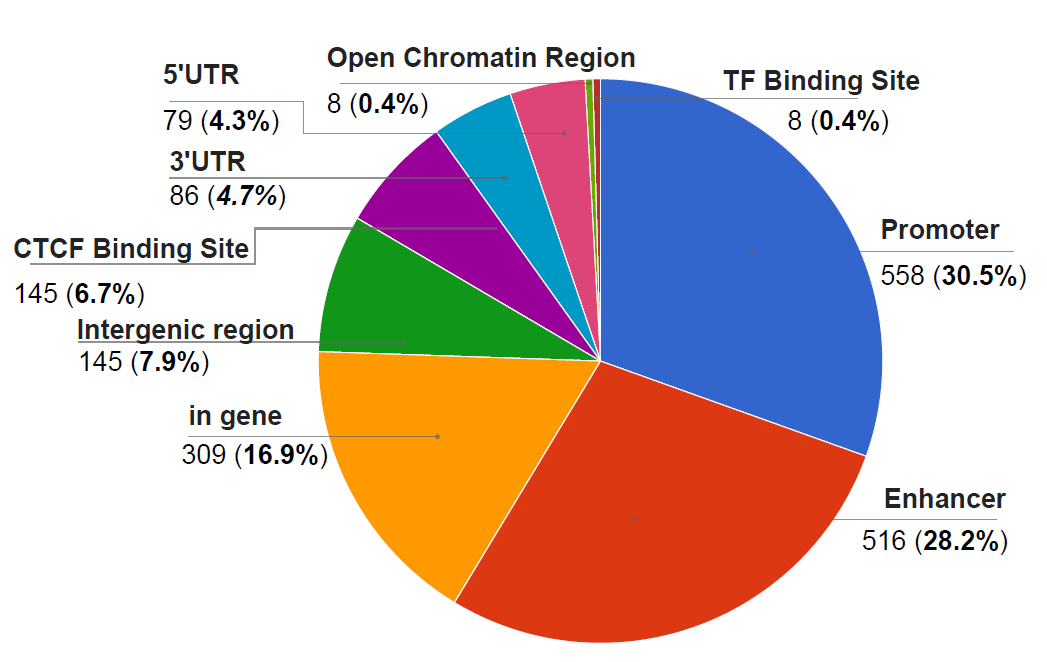


**Supplementary Fig 11.** Pie chart describing the distribution of the different genomic categories identified by ATACseq to have differential chromatin accessibility

**Supplementary Fig 12.** ChIP-PCR experiment revealed the -1000bp promoter region of Notch1 as the only significantly enriched region by chromatin immunoprecipitation using antibodies specific for H4K16Ac. This establishes the relevance of this region for the epigenetic control of Notch1 determined by SPV106 treatment.


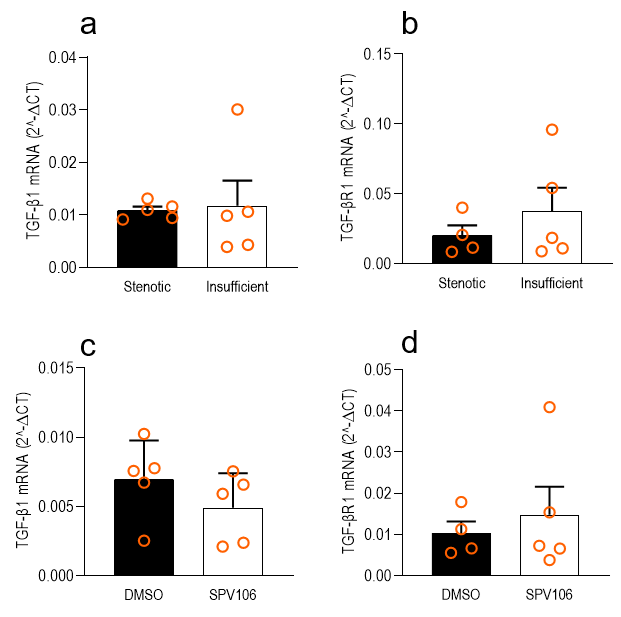


**Supplementary Fig 13.** Neither the stenotic/insufficient phenotype, nor the treatment with SPV106 affected the transcription of genes encoding for *TGF-β* or *TGF-βR1*. The *n* of biological replicates is 5 in panels **a -** **b**, and 5 in panel **a**, **c**, and 4 in panels **b**, **d**. The n of biological replicates is represented by the number of dots overlapped to each of the histogram plots, each indicating an individual cell donor.

**
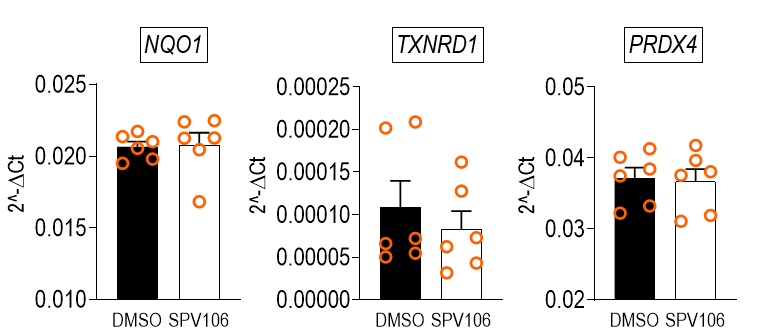
**

**Supplementary Fig 14**. RT-qPCR of genes involved in oxidative stress related to senescence. None of these genes was downregulated by SPV106 treatment.


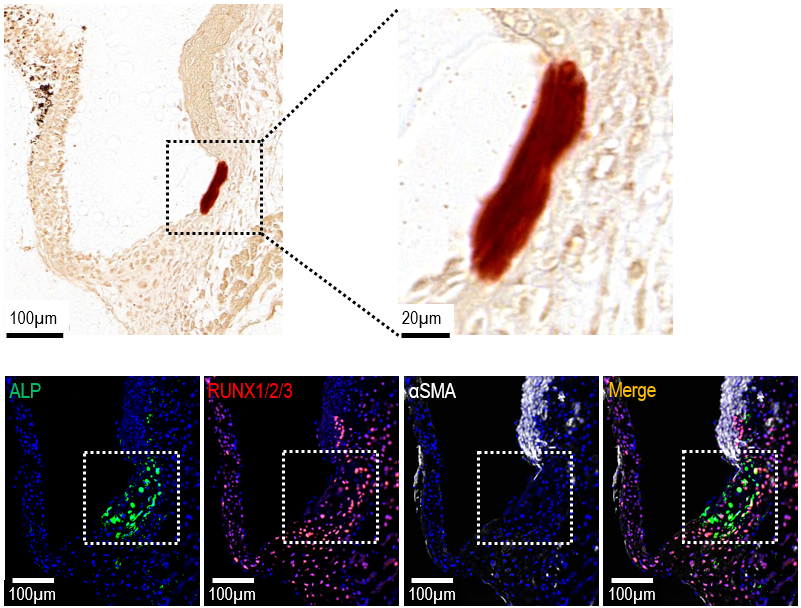


**Supplementary Fig 15.**  This panel shows the region included in Figure 6A at a higher magnification and after staining with calcific markers ALP and Runx. As shown in the figure the area of calcification coincides very sharply with the area characterized by the pathologic markers.

**
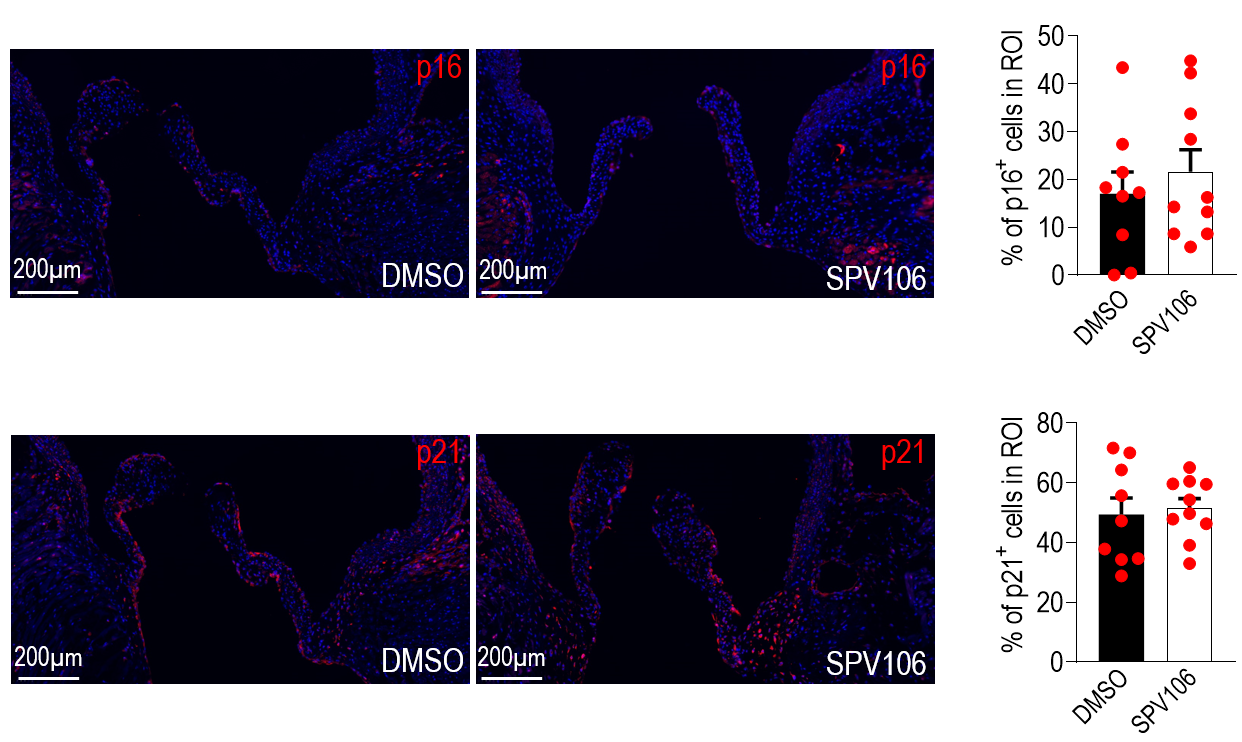
**

**Supplementary Fig 16.** Representative pictures of p16 and p21 staining of mouse aortic valves cultured for one week in the MTCS under calcifying conditions in the presence of SPV106 or DMSO (control). The bar graphs on the right side show the quantification of the percentage of the cells expressing the senescence markers compared to controls.

**
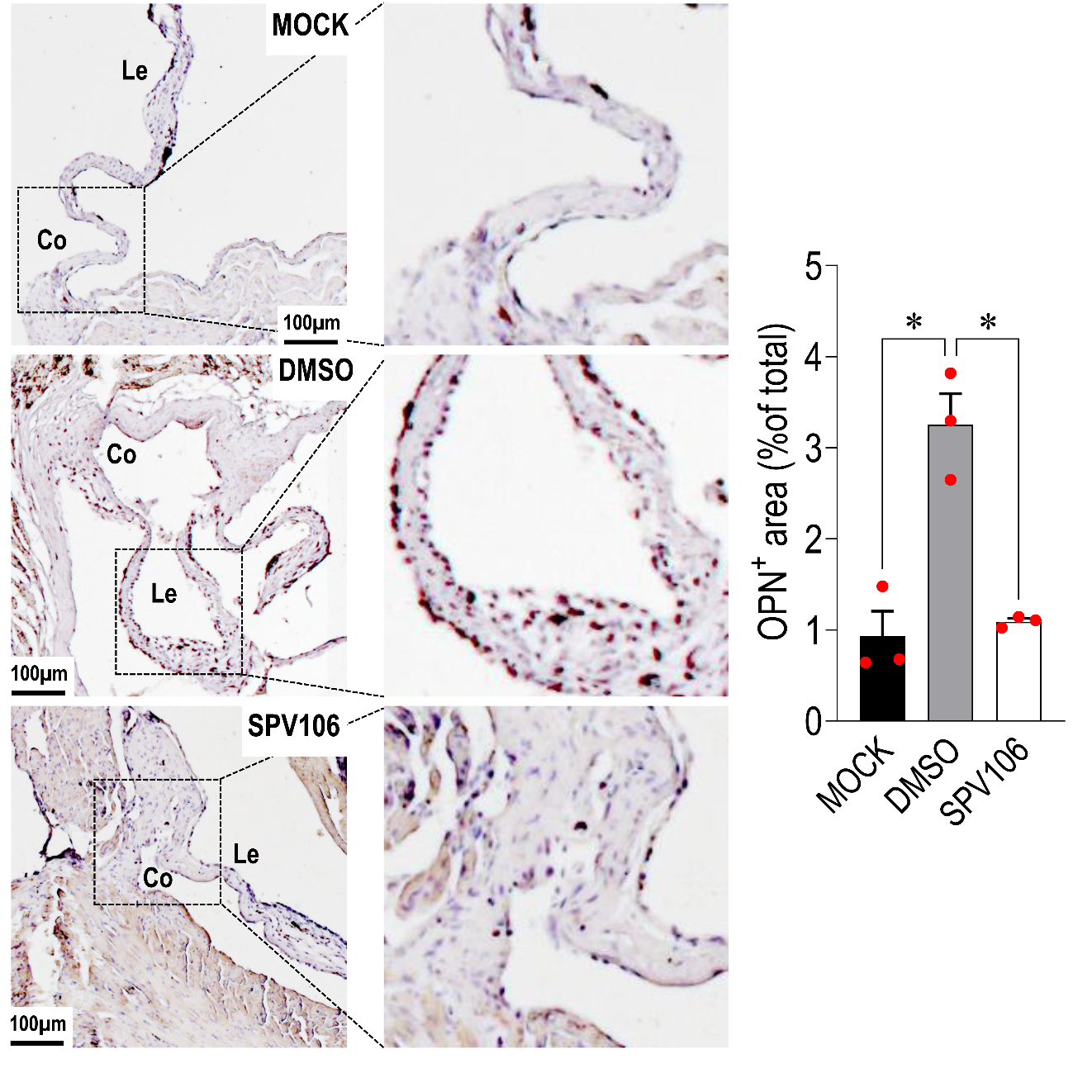
**

**Supplementary Fig 17.** OPN quantification in the aortic valves of control mice (MOCK) and mice treated with DMSO or SPV106 as described in **Figure 6**. As shown by the low and high magnification panels and the graph quantification, the presence of OPN was enhanced by Vitamin-D treatment and was reverted by SPV106 treatment. Quantification of OPN^+^ area was performed similarly to that of von Kossa staining to assess calcification in **Figure 1**.

**Table S1.** Main demographic/risk characteristics of the sVICs and iVICs donors. Age data are expressed as mean ± SD. The results of the statistical analyses to compare differences in characteristics are provided with an indication of the tests employed.

| **Variable** | **Stenosis** | **Insufficiency** | ***P*** |
| --- | --- | --- | --- |
| Age (years) | 68.1 ± 7.0 | 65.6 ± 9.5 | 0.34 (unpaired t-test) |
| **Male, *n* (%)** | **11/24 (45%)** | **15/16 (93.75%)** | **0.0022 (Fisher’s test)** |
| Hypertension, *n* (%) | 12/24 (50%) | 6/16 (37.5%) | 0.525 (Fisher’s test) |
| Dyslipidemia, *n* (%) | 8/24 (33.3%) | 2/16 (12.5%) | 0.263 (Fisher’s test) |
| Diabetes, *n* (%) | 3/24 (12.5%) | 0/16 (0%) | 0.261 (Fisher’s test) |
| BAV, *n* (%) | 5/24 (20.8%) | 3/16 (18.7%) | >0.999 (Fisher’s test) |

**Table S2.** Differentially expressed genes in iVICs vs. sVICs. The table lists gene names, normalized expression levels in sVICs and iVICs (mean ± SD; n = 4 cell lines per group), expression fold changes (FC) reported as iVICs/sVICs ratios, P-values of comparisons by unpaired t-test, and P-value adjusted for false discovery rate (q-values).

| **Genes** | **sVICs** | **iVICs** | **FC** | ***P* value** | ***q* value** |
| --- | --- | --- | --- | --- | --- |
| *PCGF3* | 1.50 ± 1.65 | 4163.29 ± 1265.47 | 2777.74 | *0.000004* | 0.00025 |
| *PHF21B* | 1.31 ± 1.10 | 887.93 ± 246.95 | 678.78 | *0.000006* | 0.00026 |
| *MTA2* | 1.05 ± 0.43 | 9.48 ± 1.67 | 9.00 | *0.000036* | 0.00121 |
| *PHC1* | 1.06 ± 0.43 | 8.49 ± 1.91 | 8.01 | *0.000088* | 0.00206 |
| *PCGF1* | 1.03 ± 0.29 | 6.58 ± 2.00 | 6.41 | *0.000072* | 0.00196 |
| *SPEN* | 1.10 ± 0.55 | 5.75 ± 1.44 | 5.21 | *0.001406* | 0.01734 |
| *PHF2* | 1.03 ± 0.32 | 5.15 ± 1.21 | 4.97 | *0.000174* | 0.00313 |
| *PHF7* | 1.04 ± 0.36 | 4.87 ± 0.75 | 4.66 | *0.000226* | 0.00353 |
| *RING1* | 1.01 ± 0.11 | 4.30 ± 1.79 | 4.27 | *0.000529* | 0.00775 |
| *MBD3* | 1.03 ± 0.29 | 3.35 ± 0.65 | 3.24 | *0.000777* | 0.01011 |
| *NSD1* | 1.12 ± 0.26 | 3.43 ± 0.93 | 3.08 | *0.001786* | 0.02039 |
| *NAB2* | 1.00 ± 0.02 | 2.60 ± 0.49 | 2.60 | *0.000075* | 0.00196 |
| *PHF5A* | 1.02 ± 0.21 | 1.90 ± 0.31 | 1.86 | *0.002517* | 0.02681 |
| *KMT5B* | 1.03 ± 0.22 | 1.86 ± 0.31 | 1.80 | *0.004175* | 0.03623 |
| *CDYL* | 1.09 ± 0.20 | 0.67 ± 0.03 | -1.63 | *0.004055* | 0.03623 |
| *PCGF2* | 1.00 ± 0.08 | 0.51 ± 0.01 | -1.97 | *0.000003* | 0.00025 |
| *PCGF6* | 1.06 ± 0.48 | 0.36 ± 0.10 | -2.94 | *0.007217* | 0.06039 |
| *SMARCA4* | 1.01 ± 0.21 | 0.32 ± 0.15 | -3.15 | *0.003169* | 0.03228 |
| *HDAC11* | 1.06 ± 0.35 | 0.30 ± 0.19 | -3.50 | *0.009062* | 0.07322 |
| *MECP2* | 1.03 ± 0.33 | 0.21 ± 0.04 | -4.86 | *0.000097* | 0.00207 |
| *WDR11* | 1.15 ± 0.71 | 0.22 ± 0.03 | -5.14 | *0.003695* | 0.03518 |
| *PCGF5* | 1.06 ± 0.44 | 0.16 ± 0.00 | -6.56 | *0.000151* | 0.00294 |
| *PHF13* | 1.06 ± 0.46 | 0.15 ± 0.06 | -7.29 | *0.000677* | 0.00934 |
| *PHF1* | 1.24 ± 0.98 | 0.16 ± 0.04 | -7.51 | *0.003754* | 0.03518 |
| *MBD4* | 1.15 ± 0.79 | 0.13 ± 0.07 | -8.82 | *0.001827* | 0.02039 |
| *MTA1* | 1.05 ± 0.36 | 0.11 ± 0.05 | -9.20 | *0,000221* | 0.00353 |
| *PHF6* | 1.08 ± 0.54 | 0.02 ± 0.01 | -50.87 | *0.000009* | 0.00037 |
| *PHF21A* | 1.06 ± 0.41 | 0.004 ± 0.001 | -284.89 | *0.000001* | 0.00018 |
| *PBRM1* | 1.16 ± 0.73 | 0.00 ± 0.00 | -373.59 | *0.000004* | 0.00025 |

**Table S3.** Differentially expressed genes in SPV106 treated *vs.* control sVICs. The table lists gene names, normalized expression levels in control (CTRL) and SPV106-treated cells (mean ± SD; n = 4 cell lines per group), expression fold changes (FC) reported as SPV106/CTRL ratios, *P*-values of pairwise comparisons by paired *t*-test, and *P*-value adjusted for false discovery rate (*q*-values).

| **Genes** | **CTRL** | **SPV106** | **FC** | ***P* value** | ***q* value** |
| --- | --- | --- | --- | --- | --- |
| *PRMT8* | 1.14 ± 0.59 | 167.54 ± 75.47 | 147.42 | *0.00176* | 0.0336 |
| *KMT2E* | 1.04 ± 0.33 | 2.77 ± 0.32 | 2.67 | *0.00380* | 0.0378 |
| *EED* | 1.01 ± 0.15 | 1.67 ± 0.24 | 1.66 | *0.00212* | 0.0341 |
| *UBE2B* | 1.01 ± 0.12 | 1.65 ± 0.23 | 1.63 | *0.02285* | 0.0783 |
| *BPTF* | 1.01 ± 0.15 | 1.60 ± 0.17 | 1.59 | *0.00080* | 0.0336 |
| *CDKN2A* | 1.06 ± 0.40 | 1.66 ± 0.33 | 1.56 | *0.02854* | 0.0852 |
| *USP16* | 1.02 ± 0.24 | 1.58 ± 0.05 | 1.54 | *0.02244* | 0.0783 |
| *IGFBP7* | 1.05 ± 0.36 | 1.62 ± 0.32 | 1.54 | *0.01116* | 0.0577 |
| *MBD2* | 1.01 ± 0.12 | 1.46 ± 0.14 | 1.45 | *0.02626* | 0.0822 |
| *ATM* | 1.03 ± 0.26 | 1.48 ± 0.31 | 1.44 | *0.02119* | 0.0783 |
| *CREG1* | 1.01 ± 0.15 | 1.42 ± 0.08 | 1.41 | *0.01209* | 0.0587 |
| *SOD1* | 1.02 ± 0.24 | 1.43 ± 0.26 | 1.40 | *0.03449* | 0.0921 |
| *TP53BP1* | 1.01 ± 0.14 | 1.39 ± 0.24 | 1.37 | *0.02960* | 0.0866 |
| *PIK3CA* | 1.00 ± 0.11 | 1.36 ± 0.21 | 1.35 | *0.02634* | 0.0822 |
| *BRD7* | 1.01 ± 0.20 | 1.35 ± 0.16 | 1.34 | *0.00988* | 0.0551 |
| *RPS6KA3* | 1.00 ± 0.12 | 1.31 ± 0.15 | 1.30 | *0.02046* | 0.0783 |
| *PHF1* | 1.00 ± 0.06 | 1.30 ± 0.28 | 1.30 | *0.04060* | 0.0998 |
| *KAT8* | 1.00 ± 0.06 | 1.29 ± 0.15 | 1.29 | *0.00667* | 0.0464 |
| *ING2* | 1.01 ± 0.18 | 1.28 ± 0.13 | 1.27 | *0.03023* | 0.0866 |
| *BMI1* | 1.00 ± 0.12 | 1.22 ± 0.09 | 1.21 | *0.02563* | 0.0822 |
| *GLB1* | 1.01 ± 0.10 | 1.18 ± 0.21 | 1.17 | *0.02731* | 0.0827 |
| *PTEN* | 1.00 ± 0.07 | 1.16 ± 0.11 | 1.16 | *0.03147* | 0.0873 |
| *MBD4* | 1.00 ± 0.07 | 1.13 ± 0.09 | 1.13 | *0.00785* | 0.0519 |
| *PCGF6* | 1.01 ± 0.18 | 0.87 ± 0.17 | -1.16 | *0.00863* | 0.0519 |
| *CDYL* | 1.01 ± 0.11 | 0.86 ± 0.07 | -1.17 | *0.02982* | 0.0866 |
| *ING1* | 1.01 ± 0.13 | 0.80 ± 0.11 | -1.25 | *0.01710* | 0.0729 |
| *HDAC1* | 1.03 ± 0.28 | 0.82 ± 0.20 | -1.26 | *0.01188* | 0.0587 |
| *AKT1* | 1.01 ± 0.21 | 0.79 ± 0.19 | -1.28 | *0.01962* | 0.0773 |
| *PRMT1* | 1.02 ± 0.20 | 0.77 ± 0.17 | -1.32 | *0.01777* | 0.0743 |
| *BAZ1B* | 1.01 ± 0.15 | 0.76 ± 0.20 | -1.33 | *0.01003* | 0.0551 |
| *NEK6* | 1.02 ± 0.26 | 0.76 ± 0.15 | -1.34 | *0.02204* | 0.0783 |
| *CHD4* | 1.00 ± 0.12 | 0.74 ± 0.16 | -1.35 | *0.00522* | 0.0454 |
| *CITED2* | 1.02 ± 0.24 | 0.75 ± 0.12 | -1.36 | *0.03859* | 0.0995 |
| *CBX4* | 1.01 ± 0.16 | 0.74 ± 0.10 | -1.36 | *0.03995* | 0.0998 |
| *RING1* | 1.00 ± 0.10 | 0.73 ± 0.14 | -1.38 | *0.01274* | 0.0592 |
| *ARD1A* | 1.00 ± 0.02 | 0.72 ± 0.12 | -1.39 | *0.02724* | 0.0827 |
| *NAB2* | 1.01 ± 0.16 | 0.72 ± 0.25 | -1.39 | *0.03707* | 0.0968 |
| *BRD1* | 1.00 ± 0.08 | 0.71 ± 0.18 | -1.41 | *0.02620* | 0.0822 |
| *CHD6* | 1.01 ± 0.14 | 0.72 ± 0.10 | -1.41 | *0.00193* | 0.0336 |
| *PBRM1* | 1.01 ± 0.18 | 0.71 ± 0.15 | -1.43 | *0.00869* | 0.0519 |
| *INO80* | 1.01 ± 0.14 | 0.70 ± 0.13 | -1.43 | *0.01571* | 0.0684 |
| *TBX3* | 1.05 ± 0.36 | 0.73 ± 0.37 | -1.44 | *0.01484* | 0.0660 |
| *SMARCA4* | 1.02 ± 0.24 | 0.71 ± 0.23 | -1.44 | *0.00353* | 0.0378 |
| *BRD2* | 1.01 ± 0.19 | 0.70 ± 0.21 | -1.45 | *0.01954* | 0.0773 |
| *PCGF2* | 1.01 ± 0.16 | 0.70 ± 0.19 | -1.45 | *0.01422* | 0.0646 |
| *MBD1* | 1.00 ± 0.08 | 0.69 ± 0.13 | -1.46 | *0.03482* | 0.0921 |
| *SPEN* | 1.01 ± 0.19 | 0.68 ± 0.15 | -1.48 | *0.02483* | 0.0822 |
| *HRAS* | 1.01 ± 0.18 | 0.68 ± 0.19 | -1.50 | *0.00570* | 0.0459 |
| *CCND1* | 1.06 ± 0.36 | 0.71 ± 0.20 | -1.50 | *0.02104* | 0.0783 |
| *CBX1* | 1.03 ± 0.31 | 0.68 ± 0.12 | -1.52 | *0.02556* | 0.0822 |
| *ING5* | 1.02 ± 0.22 | 0.67 ± 0.14 | -1.52 | *0.00925* | 0.0537 |
| *SETD1B* | 1.04 ± 0.30 | 0.67 ± 0.12 | -1.54 | *0.04014* | 0.0998 |
| *NSD1* | 1.02 ± 0.22 | 0.66 ± 0.07 | -1.55 | *0.00624* | 0.0459 |
| *CTBP2* | 1.01 ± 0.12 | 0.64 ± 0.17 | -1.56 | *0.03217* | 0.0873 |
| *E2F1* | 1.17 ± 0.76 | 0.74 ± 0.47 | -1.59 | *0.03184* | 0.0873 |
| *MBD3* | 1.01 ± 0.17 | 0.63 ± 0.07 | -1.61 | *0.00125* | 0.0336 |
| *MAP1K1* | 1.02 ± 0.25 | 0.63 ± 0.16 | -1.61 | *0.00051* | 0.0336 |
| *CTBP1* | 1.00 ± 0.06 | 0.62 ± 0.10 | -1.62 | *0.00180* | 0.0336 |
| *PHC2* | 1.00 ± 0.05 | 0.61 ± 0.08 | -1.64 | *0.00266* | 0.0348 |
| *BAZ2A* | 1.01 ± 0.16 | 0.61 ± 0.11 | -1.65 | *0.00309* | 0.0378 |
| *CARM1* | 1.07 ± 0.44 | 0.64 ± 0.25 | -1.67 | *0.02281* | 0.0783 |
| *BRPF3* | 1.00 ± 0.11 | 0.60 ± 0.06 | -1.67 | *0.00007* | 0.0142 |
| *EZH2* | 1.04 ± 0.39 | 0.61 ± 0.18 | -1.71 | *0.01132* | 0.0577 |
| *PCGF3* | 1.04 ± 0.34 | 0.60 ± 0.16 | -1.73 | *0.00341* | 0.0378 |
| *CDYL2* | 1.05 ± 0.38 | 0.60 ± 0.31 | -1.74 | *0.03152* | 0.0873 |
| *BRD3* | 1.01 ± 0.13 | 0.57 ± 0.16 | -1.75 | *0.00498* | 0.0452 |
| *BRD4* | 1.00 ± 0.10 | 0.56 ± 0.04 | -1.78 | *0.00021* | 0.0214 |
| *PHF2* | 1.01 ± 0.15 | 0.56 ± 0.12 | -1.79 | *0.00575* | 0.0459 |
| *MTA2* | 1.02 ± 0.22 | 0.57 ± 0.13 | -1.80 | *0.00180* | 0.0336 |
| *HDAC7* | 1.02 ± 0.26 | 0.56 ± 0.18 | -1.83 | *0.00116* | 0.0336 |
| *KAT2A* | 1.03 ± 0.28 | 0.55 ± 0.24 | -1.87 | *0.00369* | 0.0378 |
| *KDM5C* | 1.11 ± 0.59 | 0.58 ± 0.36 | -1.91 | *0.00256* | 0.0348 |
| *DOT1L* | 1.07 ± 0.46 | 0.55 ± 0.28 | -1.94 | *0.00162* | 0.0336 |
| *DNMT3A* | 1.03 ± 0.28 | 0.52 ± 0.18 | -1.96 | *0.00624* | 0.0459 |
| *CBX8* | 1.02 ± 0.24 | 0.51 ± 0.16 | -2.02 | *0.00831* | 0.0519 |
| *GADD45A* | 1.01 ± 0.13 | 0.47 ± 0.06 | -2.15 | *0.00637* | 0.0459 |
| *EGR1* | 1.02 ± 0.24 | 0.47 ± 0.11 | -2.17 | *0.01950* | 0.0773 |
| *DNMT1* | 1.16 ± 0.81 | 0.49 ± 0.21 | -2.37 | *0.02216* | 0.0783 |
| *AURKB* | 1.35 ± 1.30 | 0.56 ± 0.39 | -2.43 | *0.01132* | 0.0577 |
| *SMYD3* | 1.18 ± 0.87 | 0.47 ± 0.12 | -2.50 | *0.03904* | 0.0995 |
| *BRPF1* | 1.02 ± 0.21 | 0.40 ± 0.08 | -2.53 | *0.01236* | 0.0587 |
| *CBX6* | 1.01 ± 0.20 | 0.37 ± 0.17 | -2.76 | *0.00403* | 0.0382 |
| *SUV39H1* | 1.13 ± 0.68 | 0.33 ± 0.15 | -3.42 | *0.00254* | 0.0348 |
| *HDAC10* | 1.10 ± 0.51 | 0.18 ± 0.10 | -6.16 | *0.00093* | 0.0336 |
| *PHF21B* | 1.14 ± 0.61 | 0.04 ± 0.03 | -28.50 | *0.00816* | 0.0519 |

**Table S4.** Functional enrichment analysis of the SPV106-modulated mRNA signature in sVICs. The table lists the Reactome pathways enriched in differentially expressed genes reported in **Table S3**, ordered by significance level, derived from the analysis with ClueGO. Genes upregulated in the pathways are indicated in red.

| ID | | REACTOME_Pathway | | *P* Value | | % Genes | | Associated Genes Found |
| --- | --- | --- | --- | --- | --- | --- | --- | --- |
| R-HSA:3247509 | Chromatin modifying enzymes | | 2.65E-25 | | 9.85 | | BRD1, BRPF1, BRPF3, CARM1, CCND1, CHD4, DNMT3A, DOT1L, EED, EZH2, HDAC1, HDAC10, ING5, KAT2A, KAT8, KDM5C, KMT2E, MBD3, MTA2, NSD1, PBRM1, PHF2, PRMT1, SETD1B, SMARCA4, SMYD3, SUV39H1 | |
| R-HSA:4839726 | Chromatin organization | | 2.65E-25 | | 9.85 | | BRD1, BRPF1, BRPF3, CARM1, CCND1, CHD4, DNMT3A, DOT1L, EED, EZH2, HDAC1, HDAC10, ING5, KAT2A, KAT8, KDM5C, KMT2E, MBD3, MTA2, NSD1, PBRM1, PHF2, PRMT1, SETD1B, SMARCA4, SMYD3, SUV39H1 | |
| R-HSA:8943724 | Regulation of PTEN gene transcription | | 1.27E-18 | | 22.95 | | BMI1, CBX4, CBX6, CBX8, CHD4, EED, EGR1, EZH2, HDAC1; HDAC7, MBD3, MTA2, PHC2, RING1 | |
| R-HSA:6804758 | Regulation of TP53 Activity through Acetylation | | 2.14E-17 | | 36.67 | | AKT1, BRD1, BRD7, BRPF1, BRPF3, CHD4, HDAC1, ING2, ING5, MBD3, MTA2 | |
| R-HSA:6807070 | PTEN Regulation | | 4.59E-16 | | 11.43 | | AKT1, BMI1, CBX4, CBX6, CBX8, CHD4, EED, EGR1, EZH2, HDAC1, HDAC7, MBD3, MTA2, PHC2, PTEN, RING1 | |
| R-HSA:3108232 | SUMO E3 ligases SUMOylate target proteins | | 3.07E-14 | | 8.79 | | AURKB, BMI1, CBX4, CBX8, CDKN2A, CTBP1, DNMT1, DNMT3A, HDAC1, HDAC7, ING2, MBD1, PCGF2, PHC2, RING1, TP53BP1 | |
| R-HSA:2990846 | SUMOylation | | 5.13E-14 | | 8.51 | | AURKB, BMI1, CBX4, CBX8, CDKN2A, CTBP1, DNMT1, DNMT3A, HDAC1, HDAC7, ING2, MBD1, PCGF2, PHC2, RING1, TP53BP1 | |
| R-HSA:4655427 | SUMOylation of DNA methylation proteins | | 5.28E-14 | | 47.06 | | BMI1, CBX4, CBX8, DNMT1, DNMT3A, PCGF2, PHC2, RING1 | |
| R-HSA:212165 | Epigenetic regulation of gene expression | | 7.08E-13 | | 9.27 | | BAZ1B, BAZ2A, CHD4, DNMT1, DNMT3A, EED, EZH2, HDAC1, KAT2A, MBD2, MBD3, MTA2, PHF1, SUV39H1 | |
| R-HSA:1257604 | PIP3 activates AKT signaling | | 8.98E-13 | | 6.37 | | AKT1, BMI1, CBX4, CBX6, CBX8, CHD4, EED, EGR1, EZH2, HDAC1, HDAC7, MBD3, MTA2, PHC2, PIK3CA, PTEN, RING1 | |
| R-HSA:3700989 | Transcriptional Regulation by TP53 | | 1.22E-12 | | 5.21 | | AKT1, ATM, AURKB, BRD1, BRD7  BRPF1, BRPF3, CARM1, CDKN2A, CHD4, E2F1, GADD45A, HDAC1, ING2, ING5, MBD3, MTA2, PRMT1  PTEN | |
| R-HSA:5633007 | Regulation of TP53 Activity | | 1.58E-12 | | 8.75 | | AKT1, ATM, AURKB, BRD1, BRD7  BRPF1, BRPF3, CDKN2A, CHD4, HDAC1, ING2, ING5, MBD3, MTA2 | |
| R-HSA:9006925 | Intracellular signaling by second messengers | | 9.44E-12 | | 5.50 | | AKT1, BMI1, CBX4, CBX6, CBX8, CHD4, EED, EGR1, EZH2, HDAC1, HDAC7, MBD3, MTA2, PHC2, PIK3CA, PTEN, RING1 | |
| R-HSA:3899300 | SUMOylation of transcription cofactors | | 9.77E-12 | | 20.00 | | BMI1, CBX4, CBX8, CTBP1, ING2, MBD1, PCGF2, PHC2, RING1 | |
| R-HSA:2559583 | Cellular Senescence | | 4.26E-10 | | 6.53 | | ATM, BMI1, CBX4, CBX6, CBX8, CDKN2A, E2F1, EED, EZH2, IGFBP7, PHC2, RING1, RPS6KA3 | |
| R-HSA:2559580 | Oxidative Stress Induced Senescence | | 8.72E-09 | | 7.87 | | BMI, CBX4, CBX6, CBX8, CDKN2A, E2F1, EED, EZH2, PHC2  RING1 | |
| R-HSA:3214841 | PKMTs methylate histone lysines | | 1.75E-08 | | 11.27 | | DOT1L, EED, EZH2, KMT2E, NSD1, SETD1B, SMYD3, SUV39H1 | |
| R-HSA:3108214 | SUMOylation of DNA damage response and repair proteins | | 3.35E-08 | | 10.39 | | BMI1, CBX4, CBX8, CDKN2A, HDAC7, PCGF2, PHC2, RING1 | |
| R-HSA:4551638 | SUMOylation of chromatin organization proteins | | 3.67E-07 | | 9.86 | | BMI1, CBX4, CBX8, HDAC1, PCGF2, PHC2, RING1 | |
| R-HSA:4570464 | SUMOylation of RNA binding proteins | | 5.56E-07 | | 12.77 | | BMI1, CBX4, CBX8, PCGF2, PHC2  RING1 | |
| R-HSA:3214858 | RMTs methylate histone arginines | | 1.22E-05 | | 7.59 | | CARM1, CCND1, DNMT3A, PBRM1, PRMT1, SMARCA4 | |
| R-HSA:212300 | PRC2 methylates histones and DNA | | 1.11E-04 | | 6.85 | | DNMT1, DNMT3A, EED, EZH2, PHF1 | |
| R-HSA:2122947 | NOTCH1 Intracellular Domain Regulates Transcription | | 2.65E-04 | | 8.33 | | HDAC1, HDAC7, HDAC10, KAT2A | |
| R-HSA:6791312 | TP53 Regulates Transcription of Cell Cycle Genes | | 2.65E-04 | | 8.33 | | CARM1, E2F1, GADD45A, PRMT1 | |
| R-HSA:3214847 | HATs acetylate histones | | 3.24E-04 | | 4.23 | | BRD1, BRPF1, BRPF3, ING5, KAT2A, KAT8 | |
| R-HSA:3214815 | HDACs deacetylate histones | | 3.66E-04 | | 5.32 | | CHD4, HDAC1, HDAC10, MBD3, MTA2 | |
| R-HSA:2644602 | Signaling by NOTCH1 PEST Domain Mutants in Cancer | | 5.50E-04 | | 6.90 | | HDAC1, HDAC7, HDAC10, KAT2A | |
| R-HSA:2644603 | Signaling by NOTCH1 in Cancer | | 5.50E-04 | | 6.90 | | HDAC1, HDAC7, HDAC10, KAT2A | |
| R-HSA:2644606 | Constitutive Signaling by NOTCH1 PEST Domain Mutants | | 5.50E-04 | | 6.90 | | HDAC1, HDAC7, HDAC10, KAT2A | |
| R-HSA:2894858 | Signaling by NOTCH1 HD+PEST Domain Mutants in Cancer | | 5.50E-04 | | 6.90 | | HDAC1, HDAC7, HDAC10, KAT2A | |
| R-HSA:2894862 | Constitutive Signaling by NOTCH1 HD+PEST Domain Mutants | | 5.50E-04 | | 6.90 | | HDAC1, HDAC7, HDAC10, KAT2A | |

**Table S5.** Reactome Pathways encompassing loci with higher chromatin accessibility in SPV106 *vs.* DMSO treatment by ATAC-Seq (see **Figure G**).

| **Reactome ID** | **term** | **N. of Associated Genes** | | **% Associated genes** | **Associated gene** | ***P* - value** |  |
| --- | --- | --- | --- | --- | --- | --- | --- |
| R-HSA:9031628 | NGF-stimulated transcription | 10 | 25.6 | | [CHD4, EGR2, FOS, FOSB, ID3, JUNB, MEF2D, NAB1, NAB2, RRAD] | 0.0000073 | |
| R-HSA:212436 | Generic Transcription Pathway | 84 | 6.7 | | [ABL1, AKT3, ARNTL, ATR, AXIN1, BANP, BBC3, BTG2, CCNG2, CCNT2, CDK4, CEBPB, CHD4, CNOT3, COX4I1, DDIT3, E2F1, EHMT1, EPC1, ERCC2, FOS, FZR1, GATA4, GTF2H1, H2AC6, H2BC5, HAND2, HDAC5, HEY1, JUNB, KMT2B, KMT5A, LAMTOR5, LMO2, MAML3, MED25, NCOR2, NELFA, NELFB, NR1H3, NR2C2AP, NR4A1, NR4A2, PDPK1, PHC3, PIN1, PIP4K2B, POLR2G, PPM1A, PPP1R13B, PPP1R13L, PRDX2, PRMT5, PSMA7, PSMB10, RARG, RBFOX3, RBM14, RBM14-RBM4, RHEB, RPA3, SIN3B, SKI, SOCS3, SOX9, SP1, TEAD2, THRB, TXN, UBE2I, YBX1, YWHAZ, ZKSCAN7, ZNF304, ZNF324B, ZNF471, ZNF547, ZNF549, ZNF606, ZNF660, ZNF705E, ZNF764, ZNF771, ZNF773] | 0.00033 | |
| R-HSA:198725 | Nuclear Events (kinase and transcription factor activation) | 10 | 16.4 | | [CHD4, EGR2, FOS, FOSB, ID3, JUNB, MEF2D, NAB1, NAB2, RRAD] | 0.00042 | |
| R-HSA:74160 | Gene expression (Transcription) | 97 | 6.4 | | [ABL1, AEBP2, AKT3, ARNTL, ATR, AXIN1, BANP, BBC3, BCDIN3D, BTG2, CCNG2, CCNT2, CD3EAP, CDK4, CEBPB, CHD4, CNOT3, COX4I1, DDIT3, E2F1, EHMT1, EPC1, ERCC2, FOS, FZR1, GATA4, GTF2E1, GTF2H1, H2AC6, H2BC5, HAND2, HDAC5, HEY1, INTS6, JARID2, JUNB, KMT2B, KMT5A, LAMTOR5, LMO2, MAML3, MED25, MLLT1, MTA1, NCOR2, NELFA, NELFB, NR1H3, NR2C2AP, NR4A1, NR4A2, NUP54, PDPK1, PHC3, PIN1, PIP4K2B, POLR2G, POLR3GL, PPM1A, PPP1R13B, PPP1R13L, PRDX2, PRMT5, PSMA7, PSMB10, RARG, RBFOX3, RBM14, RBM14-RBM4, RHEB, RPA3, RPRD2, RRN3, SIN3B, SKI, SOCS3, SOX9, SP1, TEAD2, THRB, TXN, U2AF1L4, UBE2I, YBX1, YWHAZ, ZKSCAN7, ZNF304, ZNF324B, ZNF471, ZNF547, ZNF549, ZNF606, ZNF660, ZNF705E, ZNF764, ZNF771, ZNF773] | 0.00058 | |
| R-HSA:5696395 | Formation of Incision Complex in GG-NER | 8 | 18.6 | | [ERCC1, ERCC2, GTF2H1, PIAS1, RAD23B, RPA3, UBE2I, XPC] | 0.00066 | |
| R-HSA:73857 | RNA Polymerase II Transcription | 89 | 6.4 | | [ABL1, AKT3, ARNTL, ATR, AXIN1, BANP, BBC3, BTG2, CCNG2, CCNT2, CDK4, CEBPB, CHD4, CNOT3, COX4I1, DDIT3, E2F1, EHMT1, EPC1, ERCC2, FOS, FZR1, GATA4, GTF2E1, GTF2H1, H2AC6, H2BC5, HAND2, HDAC5, HEY1, INTS6, JUNB, KMT2B, KMT5A, LAMTOR5, LMO2, MAML3, MED25, MLLT1, NCOR2, NELFA, NELFB, NR1H3, NR2C2AP, NR4A1, NR4A2, PDPK1, PHC3, PIN1, PIP4K2B, POLR2G, PPM1A, PPP1R13B, PPP1R13L, PRDX2, PRMT5, PSMA7, PSMB10, RARG, RBFOX3, RBM14, RBM14-RBM4, RHEB, RPA3, RPRD2, SIN3B, SKI, SOCS3, SOX9, SP1, TEAD2, THRB, TXN, U2AF1L4, UBE2I, YBX1, YWHAZ, ZKSCAN7, ZNF304, ZNF324B, ZNF471, ZNF547, ZNF549, ZNF606, ZNF660, ZNF705E, ZNF764, ZNF771, ZNF773] | 0.00073 | |
| R-HSA:3700989 | Transcriptional Regulation by TP53 | 30 | 8.3 | | [AKT3, ATR, BANP, BBC3, BTG2, CCNT2, CHD4, CNOT3, COX4I1, E2F1, EHMT1, ERCC2, FOS, GTF2H1, KMT5A, LAMTOR5, NELFA, NELFB, PDPK1, PIN1, PIP4K2B, POLR2G, PPP1R13B, PPP1R13L, PRDX2, PRMT5, RHEB, RPA3, TXN, YWHAZ] | 0.0019 | |
| R-HSA:187037 | Signaling by NTRK1 (TRKA) | 13 | 11.3 | | [AP2S1, CHD4, DNAL4, EGR2, FOS, FOSB, ID3, IRS2, JUNB, MEF2D, NAB1, NAB2, RRAD] | 0.0023 | |
| R-HSA:6804759 | Regulation of TP53 Activity through Association with Co-factors | 4 | 28.6 | | [AKT3, BANP, PPP1R13B, PPP1R13L] | 0.0031 | |
| R-HSA:9735871 | SARS-CoV-1 targets host intracellular signalling and regulatory pathways | 4 | 26.7 | | [PDPK1, SP1, UBE2I, YWHAZ] | 0.0040 | |
| R-HSA:400253 | Circadian Clock | 9 | 12.9 | | [ARNTL, CLOCK, CRY1, CRY2, CSNK1D, DBP, KLF15, MEF2D, RBM4] | 0.0046 | |
| R-HSA:166520 | Signaling by NTRKs | 14 | 10.4 | | [AP2S1, CHD4, DNAL4, EGR2, FOS, FOSB, ID3, IRS2, JUNB, MEF2D, NAB1, NAB2, NELFB, RRAD] | 0.0051 | |
| R-HSA:6796648 | TP53 Regulates Transcription of DNA Repair Genes | 8 | 12.9 | | [ATR, CCNT2, ERCC2, FOS, GTF2H1, NELFA, NELFB, POLR2G] | 0.0072 | |
| R-HSA:2173793 | Transcriptional activity of SMAD2/SMAD3:SMAD4 heterotrimer | 7 | 13.7 | | [CCNT2, JUNB, NCOR2, PPM1A, SKI, SP1, YBX1] | 0.0083 | |
| R-HSA:383280 | Nuclear Receptor transcription pathway | 7 | 13.5 | | [NCOR2, NR1H3, NR2C2AP, NR4A1, NR4A2, RARG, THRB] | 0.0093 | |
| R-HSA:525793 | Myogenesis | 5 | 16.7 | | [ABL1, BORCS8-MEF2B, CDH15, MEF2D, SPAG9] | 0.011 | |
| R-HSA:4090294 | SUMOylation of intracellular receptors | 5 | 16.7 | | [NR1H3, NR4A2, PIAS1, THRB, UBE2I] | 0.011 | |
| R-HSA:114452 | Activation of BH3-only proteins | 5 | 16.7 | | [AKT3, BBC3, E2F1, PPP1R13B, YWHAZ] | 0.011 | |
| R-HSA:9006934 | Signaling by Receptor Tyrosine Kinases | 36 | 6.9 | | [ADAP1, AKT3, AP2S1, ATP6V1E1, ATP6V1E2, BCAR1, CHD4, COL4A2, COL5A1, DNAL4, EGR2, FGFR1, FGFR3, FOS, FOSB, ID3, IRS2, ITPR2, JUNB, LAMA5, LAMB2, MEF2D, MXD4, NAB1, NAB2, NELFB, NRG2, PDPK1, POLR2G, PSENEN, PTBP1, PTK2B, RRAD, SH3GL1, STAT6, THBS2] | 0.014 | |
| R-HSA:167152 | Formation of HIV elongation complex in the absence of HIV Tat | 6 | 13.3 | | [CCNT2, ERCC2, GTF2H1, NELFA, NELFB, POLR2G] | 0.016 | |
| R-HSA:112382 | Formation of RNA Pol II elongation complex | 7 | 12.1 | | [CCNT2, ERCC2, GTF2H1, MLLT1, NELFA, NELFB, POLR2G] | 0.016 | |
| R-HSA:75955 | RNA Polymerase II Transcription Elongation | 7 | 12.1 | | [CCNT2, ERCC2, GTF2H1, MLLT1, NELFA, NELFB, POLR2G] | 0.016 | |
| R-HSA:2559585 | Oncogene Induced Senescence | 5 | 15.2 | | [CDK4, CDKN2C, E2F1, ERF, SP1] | 0.016 | |
| R-HSA:113418 | Formation of the Early Elongation Complex | 5 | 15.2 | | [ERCC2, GTF2H1, NELFA, NELFB, POLR2G] | 0.016 | |
| R-HSA:167158 | Formation of the HIV-1 Early Elongation Complex | 5 | 15.2 | | [ERCC2, GTF2H1, NELFA, NELFB, POLR2G] | 0.016 | |
| R-HSA:8862803 | Deregulated CDK5 triggers multiple neurodegenerative pathways in Alzheimer's disease models | 4 | 18.2 | | [CDC25B, GOLGA2, LMNA, PRDX2] | 0.017 | |
| R-HSA:8863678 | Neurodegenerative Diseases | 4 | 18.2 | | [CDC25B, GOLGA2, LMNA, PRDX2] | 0.017 | |
| R-HSA:8854214 | TBC/RABGAPs | 6 | 13.0 | | [RAB11B, RAB33A, RAB5B, RAB7A, RABGAP1, ULK1] | 0.018 | |
| R-HSA:73762 | RNA Polymerase I Transcription Initiation | 6 | 12.8 | | [CD3EAP, CHD4, ERCC2, GTF2H1, MTA1, RRN3] | 0.020 | |
| R-HSA:8943724 | Regulation of PTEN gene transcription | 7 | 11.5 | | [ATN1, CHD4, HDAC5, LAMTOR5, MTA1, PHC3, RHEB] | 0.021 | |
| R-HSA:72172 | mRNA Splicing | 16 | 8.3 | | [CCAR1, CWC25, DDX42, DNAJC8, HNRNPU, LSM3, LSM4, POLR2G, PPIH, PRPF40A, PTBP1, SNRNP27, U2AF1L4, XAB2, YBX1, ZMAT5] | 0.022 | |
| R-HSA:1474290 | Collagen formation | 9 | 10.0 | | [COL16A1, COL4A2, COL5A1, COLGALT1, LOXL3, P4HA3, PLEC, PLOD2, SERPINH1] | 0.022 | |
| R-HSA:5674400 | Constitutive Signaling by AKT1 E17K in Cancer | 4 | 16.0 | | [AKT3, GSK3A, NR4A1, PDPK1] | 0.026 | |
| R-HSA:9734009 | Defective Intrinsic Pathway for Apoptosis | 4 | 16.0 | | [CDC25B, GOLGA2, LMNA, PRDX2] | 0.026 | |
| R-HSA:9007101 | Rab regulation of trafficking | 11 | 8.9 | | [AKT3, MADD, MON1B, RAB10, RAB11B, RAB33A, RAB5B, RAB7A, RABGAP1, TRAPPC10, ULK1] | 0.030 | |
| R-HSA:72163 | mRNA Splicing - Major Pathway | 15 | 8.1 | | [CCAR1, CWC25, DDX42, DNAJC8, HNRNPU, LSM3, LSM4, POLR2G, PPIH, PRPF40A, PTBP1, SNRNP27, U2AF1L4, XAB2, YBX1] | 0.031 | |
| R-HSA:674695 | RNA Polymerase II Pre-transcription Events | 8 | 9.9 | | [CCNT2, ERCC2, GTF2E1, GTF2H1, MLLT1, NELFA, NELFB, POLR2G] | 0.032 | |
| R-HSA:1368108 | BMAL1:CLOCK,NPAS2 activates circadian gene expression | 4 | 14.8 | | [ARNTL, CLOCK, DBP, KLF15] | 0.033 | |
| R-HSA:1650814 | Collagen biosynthesis and modifying enzymes | 7 | 10.4 | | [COL16A1, COL4A2, COL5A1, COLGALT1, P4HA3, PLOD2, SERPINH1] | 0.034 | |
| R-HSA:8848021 | Signaling by PTK6 | 6 | 11.1 | | [BCAR1, CDK4, EPAS1, NRG2, SOCS3, STAP2] | 0.037 | |
| R-HSA:9006927 | Signaling by Non-Receptor Tyrosine Kinases | 6 | 11.1 | | [BCAR1, CDK4, EPAS1, NRG2, SOCS3, STAP2] | 0.037 | |
| R-HSA:5696398 | Nucleotide Excision Repair | 10 | 9.0 | | [ERCC1, ERCC2, GTF2H1, PIAS1, POLR2G, RAD23B, RPA3, UBE2I, XAB2, XPC] | 0.037 | |
| R-HSA:9619665 | EGR2 and SOX10-mediated initiation of Schwann cell myelination | 4 | 14.3 | | [EGR2, NAB1, NAB2, SREBF2] | 0.038 | |
| R-HSA:429914 | Deadenylation-dependent mRNA decay | 6 | 10.9 | | [CNOT3, EIF4A1, LSM3, LSM4, SKIV2L, TUT4] | 0.040 | |
| R-HSA:1655829 | Regulation of cholesterol biosynthesis by SREBP (SREBF) | 6 | 10.9 | | [KPNB1, MVD, MVK, SCD, SP1, SREBF2] | 0.040 | |
| R-HSA:109606 | Intrinsic Pathway for Apoptosis | 6 | 10.9 | | [AKT3, BBC3, CARD8, E2F1, PPP1R13B, YWHAZ] | 0.040 | |
| R-HSA:167172 | Transcription of the HIV genome | 7 | 10.0 | | [CCNT2, ERCC2, GTF2E1, GTF2H1, NELFA, NELFB, POLR2G] | 0.041 | |
| R-HSA:2426168 | Activation of gene expression by SREBF (SREBP) | 5 | 11.9 | | [MVD, MVK, SCD, SP1, SREBF2] | 0.042 | |
| R-HSA:167169 | HIV Transcription Elongation | 5 | 11.6 | | [ERCC2, GTF2H1, NELFA, NELFB, POLR2G] | 0.046 | |
| R-HSA:167200 | Formation of HIV-1 elongation complex containing HIV-1 Tat | 5 | 11.6 | | [ERCC2, GTF2H1, NELFA, NELFB, POLR2G] | 0.046 | |
| R-HSA:167246 | Tat-mediated elongation of the HIV-1 transcript | 5 | 11.6 | | [ERCC2, GTF2H1, NELFA, NELFB, POLR2G] | 0.046 | |
| R-HSA:1169408 | ISG15 antiviral mechanism | 7 | 9.7 | | [EIF4A1, KPNA2, KPNB1, MX1, MX2, NUP54, PIN1] | 0.047 | |
| R-HSA:909733 | Interferon alpha/beta signaling | 7 | 9.7 | | [HLA-F, IFI35, IRF6, KPNB1, MX1, MX2, SOCS3] | 0.047 | |
| R-HSA:6804758 | Regulation of TP53 Activity through Acetylation | 4 | 13.3 | | [AKT3, CHD4, PIN1, PIP4K2B] | 0.047 | |
| R-HSA:109581 | Apoptosis | 14 | 7.8 | | [AKT3, BBC3, CARD8, E2F1, KPNB1, LMNA, OPA1, PLEC, PPP1R13B, PSMA7, PSMB10, STK24, UNC5B, YWHAZ] | 0.047 | |
| R-HSA:2644602 | Signaling by NOTCH1 PEST Domain Mutants in Cancer | 6 | 10.3 | | [HDAC5, HEY1, HEYL, MAML3, NCOR2, PSENEN] | 0.049 | |
| R-HSA:2644603 | Signaling by NOTCH1 in Cancer | 6 | 10.3 | | [HDAC5, HEY1, HEYL, MAML3, NCOR2, PSENEN] | 0.049 | |
| R-HSA:2644606 | Constitutive Signaling by NOTCH1 PEST Domain Mutants | 6 | 10.3 | | [HDAC5, HEY1, HEYL, MAML3, NCOR2, PSENEN] | 0.049 | |
| R-HSA:2894858 | Signaling by NOTCH1 HD+PEST Domain Mutants in Cancer | 6 | 10.3 | | [HDAC5, HEY1, HEYL, MAML3, NCOR2, PSENEN] | 0.049 | |
| R-HSA:2894862 | Constitutive Signaling by NOTCH1 HD+PEST Domain Mutants | 6 | 10.3 | | [HDAC5, HEY1, HEYL, MAML3, NCOR2, PSENEN] | 0.049 | |

**Table S6.** Reactome Pathways encompassing loci with lower chromatin accessibility in SPV106 *vs.* DMSO treatment by ATAC-Seq (see **Figure 5H**).

| **Reactome ID** | **term** | **N. of Associated genes** | **% Associated genes** | **Associated gene** | ***P* - value** |
| --- | --- | --- | --- | --- | --- |
| R-HSA:9662360 | Sensory processing of sound by inner hair cells of the cochlea | 12 | 17.4 | [ACTG1, ATP2B2, CAPZB, FSCN2, GRXCR2, KCNMA1, MSN, PCLO, SPTBN1, TMIE, TRIOBP, USH1C] | 0.0000047 |
| R-HSA:9659379 | Sensory processing of sound | 12 | 15.8 | [ACTG1, ATP2B2, CAPZB, FSCN2, GRXCR2, KCNMA1, MSN, PCLO, SPTBN1, TMIE, TRIOBP, USH1C] | 0.000013 |
| R-HSA:9662361 | Sensory processing of sound by outer hair cells of the cochlea | 10 | 18.5 | [ACTG1, ATP2B2, FSCN2, GRXCR2, KCNMA1, MSN, SPTBN1, TMIE, TRIOBP, USH1C] | 0.000016 |
| R-HSA:2682334 | EPH-Ephrin signaling | 13 | 14.1 | [ACTG1, ARHGEF7, ARPC1A, EFNA3, EFNA4, EFNB1, EPHB3, KALRN, LIMK2, MMP2, PSEN1, PTK2, RAC1] | 0.000020 |
| R-HSA:9012999 | RHO GTPase cycle | 34 | 7.6 | [ACTG1, ACTN1, ADD3, ANKFY1, ARHGAP17, ARHGAP24, ARHGAP42, ARHGDIA, ARHGEF10L, ARHGEF17, ARHGEF25, ARHGEF3, ARHGEF7, BAIAP2L1, BCR, CAPZB, CHN2, CYFIP1, CYFIP2, DOCK5, DST, FGD4, FLOT1, FMNL2, GIT2, HMOX2, KALRN, MCF2L, PLXNA1, RAC1, RAPGEF1, SPTBN1, SRGAP1, TAOK3] | 0.000034 |
| R-HSA:1474244 | Extracellular matrix organization | 25 | 8.3 | [ACTN1, ADAM15, ADAMTS8, BMP2, CAPN2, CD44, COL15A1, COL6A2, COL6A3, COL8A1, COL8A2, DDR1, DST, HTRA1, ITGA3, LTBP2, MMP15, MMP2, P3H2, P4HB, PECAM1, PSEN1, SERPINE1, TIMP2, TNC] | 0.000080 |
| R-HSA:3928662 | EPHB-mediated forward signaling | 8 | 19.0 | [ACTG1, ARPC1A, EFNB1, EPHB3, KALRN, LIMK2, PTK2, RAC1] | 0.000094 |
| R-HSA:8951671 | RUNX3 regulates YAP1-mediated transcription | 4 | 50.0 | [RUNX3, TEAD1, TEAD4, WWTR1] | 0.000097 |
| R-HSA:9013149 | RAC1 GTPase cycle | 18 | 9.7 | [ARHGAP17, ARHGAP24, ARHGAP42, ARHGDIA, ARHGEF25, ARHGEF7, BAIAP2L1, BCR, CHN2, CYFIP1, CYFIP2, DOCK5, GIT2, KALRN, MCF2L, RAC1, SRGAP1, TAOK3] | 0.000098 |
| R-HSA:162582 | Signal Transduction | 123 | 4.8 | [ACTG1, ACTN1, ADCY7, ADCY9, ADD3, ADRA1D, AKT1, ANKFY1, ARHGAP17, ARHGAP24, ARHGAP42, ARHGDIA, ARHGEF10L, ARHGEF17, ARHGEF25, ARHGEF3, ARHGEF37, ARHGEF7, ARPC1A, BAIAP2L1, BCR, BMP2, CAMK2A, CAPZB, CBFB, CBX6, CCL2, CDC14A, CHN2, COL6A2, COL6A3, CSNK1A1, CXCL13, CYFIP1, CYFIP2, DNM3, DOCK5, DST, DYNC1I1, ELMO1, EPS15, ESR1, FGD4, FGF1, FGF20, FLOT1, FMNL2, FOXH1, GIT2, GPC5, GTF2F2, HMOX2, IFT122, IL1RAP, IL1RL1, INSR, ITGA3, KALRN, KDM4A, KDM4B, KHDRBS1, LAMTOR2, LATS1, LGR5, LGR6, LIMK2, LTBP2, MAPKAPK2, MAPKAPK3, MCF2L, MLST8, MMP2, NEDD4L, NOTCH2, OPRL1, P4HB, PAG1, PBX1, PDE10A, PDE6G, PDGFRA, PDK2, PLCB1, PLXNA1, PML, POLR2B, PRKAB2, PRKCE, PSEN1, PSMC5, PTK2, RAC1, RAPGEF1, RBPJ, RGS12, RGS19, RGS20, RHO, ROR1, RUNX3, RUVBL1, SCMH1, SEH1L, SERPINE1, SFN, SMAD6, SPRED2, SPTBN1, SREBF1, SRGAP1, SSTR1, STK3, TAOK3, TJP1, TMED2, TRAK1, TSHR, UBE2L3, UHMK1, USP34, WNT2, WWTR1, ZDHHC7] | 0.00019 |
| R-HSA:193648 | NRAGE signals death through JNK | 9 | 15.3 | [ARHGEF10L, ARHGEF17, ARHGEF3, ARHGEF37, ARHGEF7, FGD4, KALRN, MCF2L, RAC1] | 0.00021 |
| R-HSA:204998 | Cell death signalling via NRAGE, NRIF and NADE | 10 | 13.2 | [ARHGEF10L, ARHGEF17, ARHGEF3, ARHGEF37, ARHGEF7, FGD4, KALRN, MCF2L, PSEN1, RAC1] | 0.00033 |
| R-HSA:3928665 | EPH-ephrin mediated repulsion of cells | 8 | 15.7 | [ACTG1, EFNA3, EFNA4, EFNB1, EPHB3, MMP2, PSEN1, RAC1] | 0.00039 |
| R-HSA:1474228 | Degradation of the extracellular matrix | 14 | 10.0 | [ADAM15, ADAMTS8, CAPN2, CD44, COL15A1, COL6A2, COL6A3, COL8A1, COL8A2, HTRA1, MMP15, MMP2, PSEN1, TIMP2] | 0.00044 |
| R-HSA:193704 | p75 NTR receptor-mediated signalling | 11 | 11.3 | [ARHGDIA, ARHGEF10L, ARHGEF17, ARHGEF3, ARHGEF37, ARHGEF7, FGD4, KALRN, MCF2L, PSEN1, RAC1] | 0.00062 |
| R-HSA:8980692 | RHOA GTPase cycle | 14 | 9.5 | [ARHGAP24, ARHGAP42, ARHGDIA, ARHGEF10L, ARHGEF17, ARHGEF25, ARHGEF3, ARHGEF7, BCR, FLOT1, HMOX2, KALRN, MCF2L, SRGAP1] | 0.00071 |
| R-HSA:9664407 | Parasite infection | 8 | 13.6 | [ACTG1, ARPC1A, CD247, CYFIP1, CYFIP2, ELMO1, PTK2, RAC1] | 0.0011 |
| R-HSA:9664417 | Leishmania phagocytosis | 8 | 13.6 | [ACTG1, ARPC1A, CD247, CYFIP1, CYFIP2, ELMO1, PTK2, RAC1] | 0.0011 |
| R-HSA:9664422 | FCGR3A-mediated phagocytosis | 8 | 13.6 | [ACTG1, ARPC1A, CD247, CYFIP1, CYFIP2, ELMO1, PTK2, RAC1] | 0.0011 |
| R-HSA:2029482 | Regulation of actin dynamics for phagocytic cup formation | 8 | 13.1 | [ACTG1, ARPC1A, CD247, CYFIP1, CYFIP2, ELMO1, PTK2, RAC1] | 0.0013 |
| R-HSA:5663213 | RHO GTPases Activate WASPs and WAVEs | 6 | 16.7 | [ACTG1, ARPC1A, CYFIP1, CYFIP2, PTK2, RAC1] | 0.0015 |
| R-HSA:416482 | G alpha (12/13) signalling events | 9 | 11.3 | [ADRA1D, ARHGEF10L, ARHGEF17, ARHGEF3, ARHGEF37, ARHGEF7, FGD4, KALRN, MCF2L] | 0.0020 |
| R-HSA:9716542 | Signaling by Rho GTPases, Miro GTPases and RHOBTB3 | 41 | 5.7 | [ACTG1, ACTN1, ADD3, ANKFY1, ARHGAP17, ARHGAP24, ARHGAP42, ARHGDIA, ARHGEF10L, ARHGEF17, ARHGEF25, ARHGEF3, ARHGEF7, ARPC1A, BAIAP2L1, BCR, CAPZB, CHN2, CYFIP1, CYFIP2, DOCK5, DST, DYNC1I1, FGD4, FLOT1, FMNL2, GIT2, HMOX2, KALRN, LIMK2, MCF2L, PLXNA1, PTK2, RAC1, RAPGEF1, SEH1L, SFN, SPTBN1, SRGAP1, TAOK3, TRAK1] | 0.0024 |
| R-HSA:4420097 | VEGFA-VEGFR2 Pathway | 10 | 10.1 | [ACTG1, AKT1, CYFIP1, CYFIP2, ELMO1, MAPKAPK2, MAPKAPK3, MLST8, PTK2, RAC1] | 0.0026 |
| R-HSA:194315 | Signaling by Rho GTPases | 40 | 5.7 | [ACTG1, ACTN1, ADD3, ANKFY1, ARHGAP17, ARHGAP24, ARHGAP42, ARHGDIA, ARHGEF10L, ARHGEF17, ARHGEF25, ARHGEF3, ARHGEF7, ARPC1A, BAIAP2L1, BCR, CAPZB, CHN2, CYFIP1, CYFIP2, DOCK5, DST, DYNC1I1, FGD4, FLOT1, FMNL2, GIT2, HMOX2, KALRN, LIMK2, MCF2L, PLXNA1, PTK2, RAC1, RAPGEF1, SEH1L, SFN, SPTBN1, SRGAP1, TAOK3] | 0.0030 |
| R-HSA:446353 | Cell-extracellular matrix interactions | 4 | 22.2 | [ACTG1, ACTN1, LIMS1, PARVA] | 0.0032 |
| R-HSA:2029480 | Fcgamma receptor (FCGR) dependent phagocytosis | 9 | 10.5 | [ACTG1, ARPC1A, CD247, CYFIP1, CYFIP2, ELMO1, PRKCE, PTK2, RAC1] | 0.0033 |
| R-HSA:3928664 | Ephrin signaling | 4 | 21.1 | [ARHGEF7, EFNB1, EPHB3, RAC1] | 0.0039 |
| R-HSA:445095 | Interaction between L1 and Ankyrins | 5 | 16.1 | [ACTG1, ANK2, ANK3, NFASC, SPTBN1] | 0.0043 |
| R-HSA:9013106 | RHOC GTPase cycle | 8 | 10.8 | [ARHGDIA, ARHGEF10L, ARHGEF17, ARHGEF25, BCR, FLOT1, FMNL2, MCF2L] | 0.0045 |
| R-HSA:2028269 | Signaling by Hippo | 4 | 20.0 | [LATS1, STK3, TJP1, WWTR1] | 0.0048 |
| R-HSA:194138 | Signaling by VEGF | 10 | 9.3 | [ACTG1, AKT1, CYFIP1, CYFIP2, ELMO1, MAPKAPK2, MAPKAPK3, MLST8, PTK2, RAC1] | 0.0049 |
| R-HSA:73887 | Death Receptor Signalling | 12 | 8.2 | [ARHGDIA, ARHGEF10L, ARHGEF17, ARHGEF3, ARHGEF37, ARHGEF7, FGD4, KALRN, MCF2L, PSEN1, RAC1, UBE2L3] | 0.0057 |
| R-HSA:5173105 | O-linked glycosylation | 10 | 9.0 | [ADAMTS8, B3GLCT, GALNT10, GALNT11, GALNT18, SEMA5A, SPON1, ST6GAL1, ST6GALNAC4, THSD4] | 0.0059 |
| R-HSA:1500931 | Cell-Cell communication | 11 | 8.5 | [ACTG1, ACTN1, CDH11, CDH13, CLDN14, DST, LIMS1, MAGI2, PARVA, PTK2, SPTBN1] | 0.0060 |
| R-HSA:392154 | Nitric oxide stimulates guanylate cyclase | 4 | 18.2 | [KCNMA1, MRVI1, NOS2, PDE10A] | 0.0068 |
| R-HSA:9675108 | Nervous system development | 33 | 5.7 | [ACTG1, ANK2, ANK3, ARHGEF7, ARPC1A, CACNA1C, COL6A2, COL6A3, DNM3, DPYSL2, EFNA3, EFNA4, EFNB1, EPHB3, KALRN, LIMK2, MMP2, MSN, NFASC, PLXNA1, PSEN1, PSMC5, PTK2, RAC1, RPL11, RPL26L1, SEMA5A, SPTBN1, SRGAP1, TEAD1, UNC5A, UTRN, WWTR1] | 0.0072 |
| R-HSA:1442490 | Collagen degradation | 7 | 10.9 | [COL15A1, COL6A2, COL6A3, COL8A1, COL8A2, MMP15, MMP2] | 0.0072 |
| R-HSA:1650814 | Collagen biosynthesis and modifying enzymes | 7 | 10.4 | [COL15A1, COL6A2, COL6A3, COL8A1, COL8A2, P3H2, P4HB] | 0.0093 |
| R-HSA:5083635 | Defective B3GALTL causes PpS | 5 | 13.5 | [ADAMTS8, B3GLCT, SEMA5A, SPON1, THSD4] | 0.0093 |
| R-HSA:216083 | Integrin cell surface interactions | 8 | 9.4 | [CD44, COL6A2, COL6A3, COL8A1, COL8A2, ITGA3, PECAM1, TNC] | 0.010 |
| R-HSA:5173214 | O-glycosylation of TSR domain-containing proteins | 5 | 13.2 | [ADAMTS8, B3GLCT, SEMA5A, SPON1, THSD4] | 0.010 |
| R-HSA:8939243 | RUNX1 interacts with co-factors whose precise effect on RUNX1 targets is not known | 5 | 13.2 | [ARID1A, CBFB, CBX6, SCMH1, SMARCD2] | 0.010 |
| R-HSA:9013026 | RHOB GTPase cycle | 7 | 10.0 | [ARHGEF10L, ARHGEF17, ARHGEF25, ARHGEF3, BCR, FLOT1, MCF2L] | 0.012 |
| R-HSA:9013405 | RHOD GTPase cycle | 6 | 11.1 | [ACTN1, ADD3, ANKFY1, ARHGAP17, CAPZB, PLXNA1] | 0.012 |
| R-HSA:9013404 | RAC2 GTPase cycle | 8 | 9.1 | [ARHGAP17, ARHGAP42, ARHGDIA, BAIAP2L1, BCR, CYFIP1, GIT2, TAOK3] | 0.013 |
| R-HSA:9013148 | CDC42 GTPase cycle | 12 | 7.7 | [ARHGAP17, ARHGAP24, ARHGAP42, ARHGDIA, ARHGEF25, ARHGEF7, BCR, FGD4, FMNL2, GIT2, MCF2L, SRGAP1] | 0.013 |
| R-HSA:6811558 | PI5P, PP2A and IER3 Regulate PI3K/AKT Signaling | 9 | 8.5 | [AKT1, ESR1, FGF1, FGF20, IL1RAP, IL1RL1, INSR, PDGFRA, RAC1] | 0.013 |
| R-HSA:1445148 | Translocation of SLC2A4 (GLUT4) to the plasma membrane | 7 | 9.7 | [ACTG1, AKT1, PRKAB2, RAC1, SFN, TBC1D1, TBC1D4] | 0.014 |
| R-HSA:1474290 | Collagen formation | 8 | 8.9 | [COL15A1, COL6A2, COL6A3, COL8A1, COL8A2, DST, P3H2, P4HB] | 0.014 |
| R-HSA:446728 | Cell junction organization | 8 | 8.8 | [ACTG1, ACTN1, CDH11, CDH13, CLDN14, DST, LIMS1, PARVA] | 0.015 |
| R-HSA:597592 | Post-translational protein modification | 67 | 4.7 | [ADAMTS8, ALG8, ALG9, ALPL, ANK2, ANK3, APOL1, ARSI, ASXL2, B3GLCT, CAPZB, COPS6, DCAF5, DDB2, DYNC1I1, ESR1, FBXO2, FBXO44, FBXW11, FEM1B, GALNT10, GALNT11, GALNT18, GFPT2, GMPPA, GRIA1, HIC1, IGFBP4, LMO7, LRRC49, LY6K, MDC1, MUL1, NPLOC4, NTM, OPCML, P4HB, PIGV, PIGY, PML, POM121, PSMC5, RAB25, RAB31, RPN2, RUVBL1, SCG3, SCMH1, SEH1L, SEMA5A, SOCS2, SPON1, SPTBN1, ST6GAL1, ST6GALNAC4, SUDS3, THSD4, TMED2, TNC, TNIP1, TRAF3, TRAPPC3, TTLL4, TULP4, UBE2L3, USP34, USP48] | 0.017 |
| R-HSA:422475 | Axon guidance | 30 | 5.4 | [ACTG1, ANK2, ANK3, ARHGEF7, ARPC1A, CACNA1C, COL6A2, COL6A3, DNM3, DPYSL2, EFNA3, EFNA4, EFNB1, EPHB3, KALRN, LIMK2, MMP2, MSN, NFASC, PLXNA1, PSEN1, PSMC5, PTK2, RAC1, RPL11, RPL26L1, SEMA5A, SPTBN1, SRGAP1, UNC5A] | 0.018 |
| R-HSA:9006925 | Intracellular signaling by second messengers | 19 | 6.1 | [ADCY7, ADCY9, AKT1, CAMK2A, CBX6, ESR1, FGF1, FGF20, IL1RAP, IL1RL1, INSR, LAMTOR2, MLST8, PDGFRA, PML, PRKCE, PSMC5, RAC1, SCMH1] | 0.018 |
| R-HSA:9013423 | RAC3 GTPase cycle | 8 | 8.5 | [ARHGAP17, ARHGAP42, BAIAP2L1, BCR, CYFIP1, GIT2, RAPGEF1, TAOK3] | 0.018 |
| R-HSA:199418 | Negative regulation of the PI3K/AKT network | 9 | 8.0 | [AKT1, ESR1, FGF1, FGF20, IL1RAP, IL1RL1, INSR, PDGFRA, RAC1] | 0.019 |
| R-HSA:8948216 | Collagen chain trimerization | 5 | 11.4 | [COL15A1, COL6A2, COL6A3, COL8A1, COL8A2] | 0.019 |
| R-HSA:2022090 | Assembly of collagen fibrils and other multimeric structures | 6 | 9.8 | [COL15A1, COL6A2, COL6A3, COL8A1, COL8A2, DST] | 0.020 |
| R-HSA:6796648 | TP53 Regulates Transcription of DNA Repair Genes | 6 | 9.7 | [DDB2, FANCC, GTF2F2, MDC1, MNAT1, POLR2B] | 0.022 |
| R-HSA:5653656 | Vesicle-mediated transport | 35 | 5.2 | [AAK1, ACTG1, AKT1, ALS2CL, ANK2, ANK3, APOL1, ARPC1A, CAPZB, COPS6, CYTH1, DENND3, DENND5B, DNM3, DYNC1I1, EPS15, GRIA1, KIFC2, LDLRAP1, PRKAB2, RAB31, RAC1, REPS1, SBF2, SCARA5, SFN, SPTBN1, SYT11, TBC1D1, TBC1D4, TJP1, TMED2, TRAPPC3, UBAP1, VPS52] | 0.023 |
| R-HSA:199991 | Membrane Trafficking | 33 | 5.2 | [AAK1, ACTG1, AKT1, ALS2CL, ANK2, ANK3, ARPC1A, CAPZB, COPS6, CYTH1, DENND3, DENND5B, DNM3, DYNC1I1, EPS15, GRIA1, KIFC2, LDLRAP1, PRKAB2, RAB31, RAC1, REPS1, SBF2, SFN, SPTBN1, SYT11, TBC1D1, TBC1D4, TJP1, TMED2, TRAPPC3, UBAP1, VPS52] | 0.026 |
| R-HSA:418597 | G alpha (z) signalling events | 5 | 10.4 | [ADCY7, ADCY9, PRKCE, RGS19, RGS20] | 0.027 |
| R-HSA:9006934 | Signaling by Receptor Tyrosine Kinases | 28 | 5.4 | [ACTG1, AKT1, ARHGEF7, COL6A2, COL6A3, CYFIP1, CYFIP2, DNM3, ELMO1, EPS15, ESR1, FGF1, FGF20, GTF2F2, INSR, ITGA3, MAPKAPK2, MAPKAPK3, MLST8, PAG1, PDGFRA, POLR2B, PRKCE, PSEN1, PTK2, RAC1, RAPGEF1, SPRED2] | 0.028 |
| R-HSA:1839126 | FGFR2 mutant receptor activation | 4 | 12.1 | [FGF1, FGF20, GTF2F2, POLR2B] | 0.028 |
| R-HSA:5663202 | Diseases of signal transduction by growth factor receptors and second messengers | 24 | 5.5 | [ACTG1, AGK, AKT1, ATG7, BCR, BIN2, CAMK2A, CSNK1A1, ESR1, FGF1, FGF20, GTF2F2, LMO7, MLST8, MSN, PDGFRA, POLR2B, PSEN1, PSMC5, RAC1, RBPJ, SPRED2, SPTBN1, TRAK1] | 0.032 |
| R-HSA:3906995 | Diseases associated with O-glycosylation of proteins | 6 | 8.8 | [ADAMTS8, B3GLCT, NOTCH2, SEMA5A, SPON1, THSD4] | 0.033 |
| R-HSA:3781865 | Diseases of glycosylation | 10 | 7.0 | [ADAMTS8, ALG8, ALG9, B3GLCT, EXT1, GPC5, NOTCH2, SEMA5A, SPON1, THSD4] | 0.036 |
| R-HSA:418346 | Platelet homeostasis | 7 | 8.0 | [ATP2B2, KCNMA1, MRVI1, NOS2, P2RX7, PDE10A, PECAM1] | 0.036 |
| R-HSA:9772573 | Late SARS-CoV-2 Infection Events | 6 | 8.6 | [ANO6, CSNK1A1, RPN2, SRPK2, ST6GAL1, ST6GALNAC4] | 0.037 |
| R-HSA:373760 | L1CAM interactions | 9 | 7.6 | [ACTG1, ANK2, ANK3, DNM3, DPYSL2, MSN, NFASC, RAC1, SPTBN1] | 0.039 |
| R-HSA:8876198 | RAB GEFs exchange GTP for GDP on RABs | 7 | 7.8 | [AKT1, ALS2CL, DENND3, DENND5B, RAB31, SBF2, TRAPPC3] | 0.040 |
| R-HSA:9013424 | RHOV GTPase cycle | 4 | 10.5 | [ARHGEF7, DST, GIT2, SPTBN1] | 0.044 |
| R-HSA:9639288 | Amino acids regulate mTORC1 | 5 | 9.1 | [LAMTOR2, MLST8, NPRL2, NPRL3, SEH1L] | 0.044 |
| R-HSA:9658195 | Leishmania infection | 11 | 6.7 | [ACTG1, ADCY7, ADCY9, ARPC1A, CD247, CYFIP1, CYFIP2, ELMO1, P2RX7, PTK2, RAC1] | 0.050 |

**Table S7. Sequence of primers used for RT-qPCR**

| Gene | Direction | Sequence 5’- 3’ |
| --- | --- | --- |
| *NOTCH-1* | Forward | CGAGGTCAACACAGACGAG |
| *NOTCH-1* | Reverse | ACAGATGGGGAGTGAAGC |
| *SOX-9* | Foward | CTGGGCAAGCTCTGGAG |
| *SOX-9* | Reverse | CGTTCTTGACCGACTTCCTC |
| *RUNX-2* | Forward | CTTCACAAATCCTCCCCAAGT |
| *RUNX-2* | Reverse | AGGCGGTCAGAGAACAAAC |
| *GAPDH* | Forward | AATCCCATCACCATCTTCCAG |
| *GAPDH* | Reverse | AAATGAGCCCCAGCCTTC |
| *HEY-1* | Forward | CAGGTAATGGAGCAAGGATCT |
| *HEY-1* | Reverse | AACTCCGATAGTCCATAGCAAG |
| *HES-1* | Forward | GAAATGACAGTGAAGCACCTC |
| *HES-1* | Reverse | TCACCTCGTTCATGCACTC |
| *HES-5* | Forward | CACCAGCCCAACTCCAAG |
| *HES-5* | Reverse | GCCTTCGCTGTAGTCCTG |
| *MYC* | Forward | TCCTCGGATTCTCTGCTCTC |
| *MYC* | Reverse | TCTTCCTCATCTTCTTGTTCCTC |
| *NQO1* | Forward | TCACCGAGAGCCTAGTTCC |
| *NQO1* | Reverse | GTGAGCCAGTACGATCAGTG |
| *TXNRD1* | Forward | GGTCATCTTCAGTAGGTCCAC |
| *TXNRD1* | Reverse | AATTCCGAGAGCGTTCCTTC |
| *PRDX4* | Forward | ACCACTCCCTGCACCTAA |
| *PRDX4* | Reverse | TCAGCTCCTTAAATTCTCCATCG |
| *OPN* | Forward | GAGGGCTTGGTTGTCAGC |
| *OPN* | Reverse | CAATTCTCATGGTAGTGAGTTTTCC |

**Data S1.** Excel file with ATAC-seq raw data. The worksheet **A** contains information about the 28862 loci with detectable reads. Worksheet **B** contains the list of the 32439 loci with >20 reads in all samples. Worksheet **C** contains the list of the of the loci with differentially opened chromatin (see Figures 5F and S9) for details.

**Supplementary References**

1 Zuccolo, E. *et al.* The microRNA-34a-Induced Senescence-Associated Secretory Phenotype (SASP) Favors Vascular Smooth Muscle Cells Calcification. Int J Mol Sci. 21, 4454 (2020).

2 Badi, I. *et al.* miR-34a Promotes Vascular Smooth Muscle Cell Calcification by Downregulating SIRT1 (Sirtuin 1) and Axl (AXL Receptor Tyrosine Kinase). Arterioscler Thromb Vasc Biol. 38, 2079-2090 (2018).

3 Ngo, D. T. *et al.* Vitamin D(2) supplementation induces the development of aortic stenosis in rabbits: interactions with endothelial function and thioredoxin-interacting protein. Eur J Pharmacol. 590, 290-296 (2008).

4 Kruithof, B. P., Lieber, S. C., Kruithof-de Julio, M., Gaussin, V. & Goumans, M. J. Culturing Mouse Cardiac Valves in the Miniature Tissue Culture System. J Vis Exp, e52750 (2015).

5 Kruithof, B. P. T. *et al.* New calcification model for intact murine aortic valves. J Mol Cell Cardiol. 156, 95-104 (2021).

6 Arif, M. *et al.* Mechanism of p300 specific histone acetyltransferase inhibition by small molecules. J Med Chem. 52, 267-277 (2009).

7 Garoffolo, G. *et al.* Reduction of Cardiac Fibrosis by Interference With YAP-Dependent Transactivation. Circ Res. 131, 239-257 (2022).

8 Zbiec-Piekarska, R. *et al.* Examination of DNA methylation status of the ELOVL2 marker may be useful for human age prediction in forensic science. Forensic science international. Genetics. 14, 161-167 (2015).

9 Chiesa, M., Colombo, G. I. & Piacentini, L. DaMiRseq-an R/Bioconductor package for data mining of RNA-Seq data: normalization, feature selection and classification. Bioinformatics. 34, 1416-1418 (2018).

10 Ritchie, M. E. *et al.* limma powers differential expression analyses for RNA-sequencing and microarray studies. Nucleic Acids Res. 43, e47 (2015).

11 Shannon, P. *et al.* Cytoscape: a software environment for integrated models of biomolecular interaction networks. Genome Res. 13, 2498-2504 (2003).

12 Bindea, G. *et al.* ClueGO: a Cytoscape plug-in to decipher functionally grouped gene ontology and pathway annotation networks. Bioinformatics. 25, 1091-1093 (2009).

13 Jassal, B. *et al.* The reactome pathway knowledgebase. Nucleic Acids Res. 48, D498-D503 (2020).

14 De Martino, S. *et al.* MALAT1 as a Regulator of the Androgen-Dependent Choline Kinase A Gene in the Metabolic Rewiring of Prostate Cancer. Cancers. 14, 2902 (2022).
